# *Helichrysum odoratissimum* (L.) Less: A Review of Its Volatile and Non-Volatile Compounds, Ethnomedicine, Pharmacological Properties and Evidence on Safety Trials in Humans

**DOI:** 10.3390/plants15081275

**Published:** 2026-04-21

**Authors:** Thanyani Emelton Ramadwa, Stephen Meddows-Taylor

**Affiliations:** Department of Life and Consumer Sciences, College of Agriculture and Environmental Sciences, Florida Campus, University of South Africa, Private Bag X6, Florida 1710, South Africa; mtayls@unisa.ac.za

**Keywords:** *Helichrysum odoratissimum*, essential oils, traditional uses, pharmacology, phytochemistry

## Abstract

*Helichrysum odoratissimum* (L.) Less. is used as a traditional medicine in South Africa to treat tuberculosis, abdominal pains, heartburn, coughs, colds, female sterility, eczema and wounds. In Uganda, the leaves are used to treat dental/oral diseases. This review aims to provide detailed information on the traditional uses, essential oils, phytochemistry, in silico studies, and pharmacological studies and propose possible future research directions on this widely investigated species. The data was gathered from various online electronic databases such as Science Direct, Scopus, Google Scholar, Web of Science, SciFinder, Wiley Online, SpringerLink, and PubMed. Reports on the essential oil composition of *H. odoratissimum* showed the dominance of monoterpenoids and sesquiterpenoid compounds. Several studies also reported the isolation of the non-volatile compounds, which were mainly flavonoids and terpenes. The species has been reported to have pharmacological activities such as antimicrobial, antimycobacterial, antioxidant activity, antidiabetic, antiproliferative, anti-inflammatory activity and antityrosinase activity. The most important study on *H. odoratissimum* was a clinical trial in human participants in South Africa addressing its in vivo irritancy potential. However, further research on the clinical and scientific aspects is needed to justify some of its other medicinal uses.

## 1. Introduction

**Figure 1:**
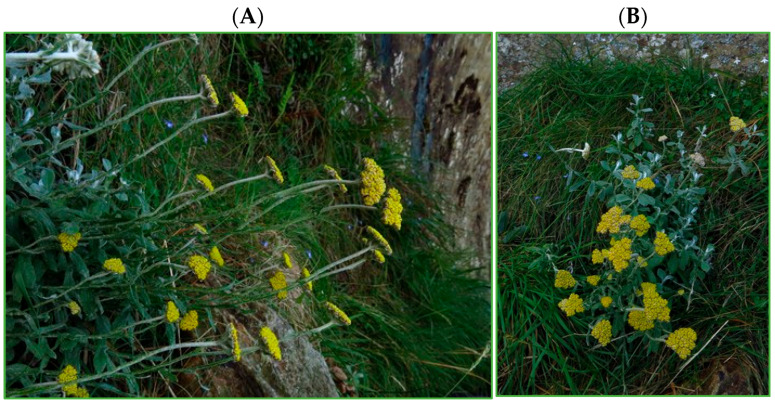
*Helichrysum odoratissimum* leaves and flower (**A**,**B**), courtesy of National Assessment: Red List of South African Plants version 2024.1. Accessed on 24 February 2026 [10].

There are over 600 *Helichrysum* species throughout the world, of which 244–250 species occur in South Africa [1,2]. The South African species are characterized by great diversity in morphology and are further divided into 30 groups [3]. This species is also widely distributed in southern Africa, in countries such as Swaziland, Mozambique, Lesotho, Botswana, Zimbabwe and Malawi [4,5]. *Helichrysum odoratissimum* (L.) Sweet, a member of the Asteraceae family and known as Imphepho in Xhosa and Zulu, is one of those native plants found in South Africa [4,5]. *H. odoratissimum,* which was also previously known as *Helichrysum bochstetteri* (Shc.-Bip. ex A. Rich.) Hook. f. and *Helichrysum bochstetteri* var *scabrum*, is a branched aromatic perennial herb with greyish-white leaves, forming large patches on grassy or rocky slopes [6,7]. The flowers, as depicted in Figure 1, are woody at the base, erect, up to 50 cm high, and bloom year-round. They are pale golden yellow, with very few flower heads carried in clusters at the terminals of the branches [4,8]. The leaves can be linear-oblong, lanceolate, lingulate, spathulate, and noticeably decurrent; the base can be narrow or broad, glandular and setose-scabrid above; the apex is often obtuse, occasionally acute, and mucronate; both sides are greyish-white and woolly, sometimes lacking wool. The scent of this plant is powerful. It is commonly used as both an insect repellent and a perfume. It is used to promote sleep and ease cramping and tense muscles [1,9].

**Figure 2:**
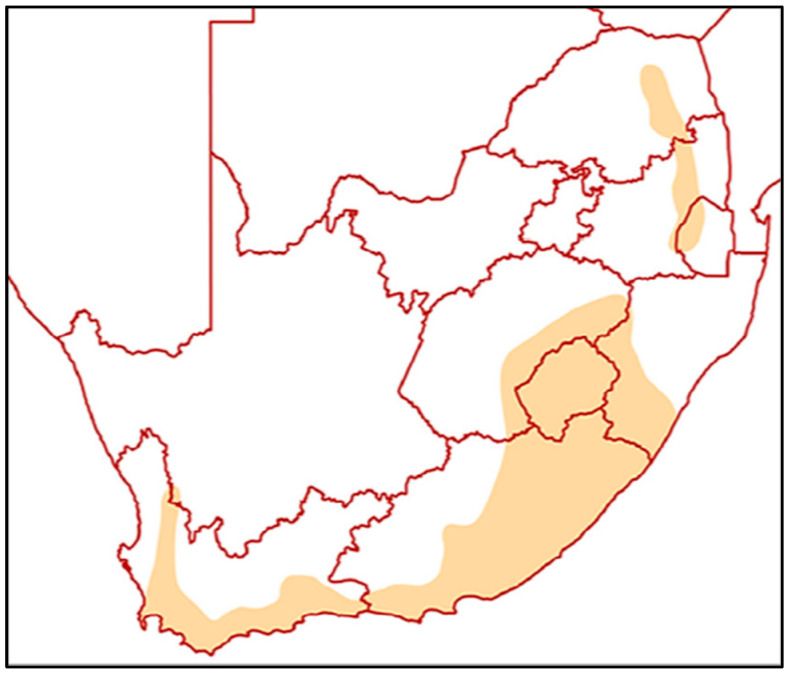
Geographical distribution of *H. odoratissimum* in South Africa as presented by [14].

*Helichrysum odoratissimum* species is found throughout the Free State, Mpumalanga, KwaZulu-Natal Province’s highlands, Limpopo Province’s Midlands, and the Soutpansberg. The species is also found in the Cape Provinces, specifically in the Eastern Cape’s coastal regions and the Cape Drakensberg highlands, as well as in the vicinity of the Cape fold mountains of Cedarberg, Giftberg in Vanrhynsdorp, and the Cape Peninsula in the Western Cape Province, South Africa as shown in Figure 2 [11,12,13]. Additionally, *H. odoratissimum* is reportedly one of the most traded species in South Africa [12].

In the Eastern Cape Province, the herb is inhaled as a cleanser and as a remedy for coughs and colds. The Zulu people, who live mostly in Kwazulu-Natal Province, utilize the leaves and stems as incense to invoke favour from the ancestors. The whole plant is used to heal wounds, dermatitis, heartburn, coughs, colds, female infertility, and abdominal problems. It is also cooked with fat and applied as a pimple treatment [6,15,16]. In Mpumalanga Province, *H. odoratissimum* leaves or entire plants have long been used to treat tuberculosis (TB) [6,17,18]. There is some empirical evidence to support the use of *H. odoratissimum* species in traditional medicines for wounds, menstrual problems, fever, catarrh, migraines, abdominal pains, indigestion, and urinary tract infections [6,12].

It is well established that the *H. odoratissimum* is abundant in volatile substances, especially essential oils. Monoterpene hydrocarbons, oxygenated monoterpenes, sesquiterpene hydrocarbons, and oxygenated sesquiterpenes are among the main classes of secondary metabolites in this species [19]. Research on the essential oil composition of *H. odoratissimum* from several African countries, including Kenya, Zimbabwe, Cameroon, Rwanda, and South Africa, revealed that monoterpenoids and sesquiterpenoids predominated as its primary constituents [20,21,22,23,24]. This species has also yielded other classes of compounds, such as 30,4′,3,5-tetrahydroxy-7-methoxyflavone, which was extracted from the acetone extract of *H. odoratissimum* flowers [25]. Van Puyvelde et al. [6] isolated helichrysetin (1-(2,4-dihyroxy-6-methoxyphenyl)-3-(4-hydroxyphenyl)-2-propen-1-one, a chalcone, and two flavonoids (3,5-dihydroxy-6,7,8-trimethoxyflavone and 3-O-methylquercetin) from the flowers of *H. odoratissimum*.

The pharmacological effects of *H. odoratissimum* have been extensively studied due to the various traditional uses of the species around the world. The antimicrobial activity of *H. odoratissimum* leaves, stems, and aerial parts was examined [26,27]. The antituberculosis action of *H. odoratissimum* is also reported in several studies [17,28]. The antiproliferative properties of *H. odoratissimum* leaves and stems have been evaluated against several cancer cell lines [29]. The anti-inflammatory activity of ethanol extracts from *H. odoratissimum* leaves and stems was evaluated using various inflammatory mechanisms/pathways, including cytokine production, cyclooxygenase, and lipoxygenase [29]. The aerial parts were tested in Uganda against *Culex quinquefasciatus*, *Anopheles gambiae*, and *Aedes aegypti* [30]. Numerous in vivo investigations have examined the toxicity of *H. odoratissimum* extracts in animal models [31]. Njagi et al. [32] reported the hypoglycaemic potential in alloxan-induced diabetic mice. Tyrosinase inhibitory effects of the essential oils from *H. odoratissimum* have also been studied [33]. The clinical research conducted in South Africa with human subjects to evaluate the potential for in vivo irritation of *H. odoratissimum* is arguably the most significant investigation on the species. Despite being categorized as a low irritant, *H. odoratissimum* had some irritant effects, which support the traditional use of plant extracts for skin-related diseases [34]. Numerous studies reviewed the phytochemistry, biological activity, and traditional applications of *Helichrysum* species [1,35]. The metabolomic profiles, pharmacological characteristics, and biological activities of *Helichrysum* species were recently examined by Adeosun and Prinsloo [36]. Considering this, the aim of this work was to present a comprehensive review of the phytochemistry, essential oils, pharmacological activities, in silico research, and traditional uses of *H. odoratissimum*.

## 2. Results

### 2.1. Ethnomedicinal Use

*H. odoratissimum* is traditionally used to treat and prevent different types of ailments across the different provinces in South Africa and other countries in the African continent, as summarized in Table 1. In Kwazulu-Natal Province, the leaves and stems are used as incense because it is said to bestow the favours of the ancestors. Healers in KwaZulu-Natal also inhale the smoke to create a trance, and it is said to be calming and beneficial for insomnia. The leaves are used to treat acne after being boiled with fat [15,37,38,39]. Other studies in South Africa reported that the leaf decoction is used as a treatment for pimples [40,41]. The whole plant is used to treat abdominal pains, heartburn, coughs, colds, female sterility, eczema and wounds [6,15,16]. In Mpumalanga Province, the leaves or whole plant parts of *H. odoratissimum* are traditionally used for the treatment of tuberculosis [17,18]. Other medicinal uses include treating respiratory ailments and nervous disorders in Cape Dutch medicine [42]. The leaves are used for sedation, cough, colds, and insecticides in Southern Africa [43]. In Uganda, the leaves are used to treat dental/oral diseases, i.e., teething syndrome. The leaves are prepared by being allowed to dry, then burned, and thereafter, rubbed on the false teeth [44,45]. *H. odoratissimum* is also used as an insect and parasite repellent, as well as perfume [46]. The leaves are used to accelerate wound healing [47]. In Lesotho, the whole plant is burnt as a fumigant for flu, coughs, colds and tuberculosis. Furthermore, the whole plant is mixed with *Olea europaea* and *Zantedeschia albomaculata* to treat backache [48,49].

### 2.2. Phytochemistry

#### 2.2.1. Volatile Compounds Identified from *H. odoratissimum*

*H. odoratissimum* is known for its aromatic properties due to the abundance of essential oils. Thus, numerous studies focused on identifying the volatile secondary metabolites from the different parts of the species collected from different locations across the African continent and other parts of the world. An overview of the volatile compounds present in *H. odoratissimum* is shown in Table 2. *H. odoratissimum* was found to contain α-Pinene (15.0%), α-humulene (13.0%), and (E)-caryophyllene (9.6%) in gas chromatography–mass spectrometry (GC-MS) analysis research carried out in Zimbabwe [20]. Kuiate et al. [21] examined essential oil samples extracted by hydrodistillation from *H. odoratissimum* leaves using gas chromatography (GC) and GC-MS. The primary components of *H. odoratissimum* leaf oil were determined to be α-pinene (47% and 41%), β-caryophyllene (14% and 5%), and α-curcumene (4% and 20%). To identify the essential oils from *H. odoratissimum*, numerous experiments have been carried out in South Africa. According to a study by Asekun et al. [24], fresh plant material included *p*-menthone (35.4%), pelugone (34.2%), and 1,8-cineole(13%). Reddy [52] found that the components of *H. odoratissimum* were (E)-Caryophyllene (9.3–25.2%), limonene (11.6–19.6%), and 1,8-cineole (11.2–17.1%). Through GC-MS analysis, Odeyemi et al. [48] discovered that the primary essential compounds from *H. odoratissimum* were limonene (14.55%), 1.8-cineole (6.56%), and α-pinene (4.20%). The Rwanda species had α-humulene (13.5%), *β*-caryophyllene (12.6%), *(Z)-β*-ocimene (10.8%) and *α*-pinene (5.7%) as the major constituents [23]. The phytochemistry of *H. odoratissimum* volatiles from leaf extracts cultivated in South Africa and hydrodistilled was examined by Lawal et al. [19] using GC and GC-MS. The study found that monoterpene hydrocarbons (72.9%), oxygenated monoterpenes (4.1%), sesquiterpene hydrocarbons (15.6%), and oxygenated sesquiterpenes (97.7%) made up most of the classes of compounds. The investigation also showed that the essential oils of *H. odoratissimum* could be categorized as α-pinene, β-pinene, limonene, α-terpinolene, linalool, α-copanene, β-elemene, α-caryophyllene, α-humulene, germacrene D, (E,E)-α-farnesene, (E)-nerolidol, caryophyllene oxide, humulene epoxide II, and drimenol. *H. odoratissimum* was found to be high in humulene (14.1%), palmitic acid (27.1%), and (E)-caryophyllene (12.6%) in Uganda [50]. Giovanelli et al. [5] found that *H. odoratissimum* species growing in Italy included α-pinene (4.11–18.39%), (E)-caryophyllene (9.67–15.85%), and 1,8-cineole (2.74–13.35%). Based on GC-MS analysis, De Canha et al. [53] determined that the terpenes caryophyllene (8.1%), α-humulene (3.9%), and α-curcumene (3.7%) were the main components of the methanolic extract of *H. odoratissimum*. The essential oils of *H. odoratissimum* had similar amounts of total monoterpenes and total sesquiterpenes (49.5% and 46.4%, respectively) [54]. Furthermore, the percentages of monoterpene hydrocarbons (23.7%), oxygenated sesquiterpenes (28.9%), and oxygenated monoterpenes (25.8%) were very similar. Furthermore, the most common compounds in each class were 1,8-cineol (25.1%), α-pinene (16.5%), and epi-cubebol (9.0%). Growing *H. odoratissimum* hydroponically, aquaponically, or in the field did not significantly change the amount of volatile chemicals it produced. A total of 116 compounds were found using all three culturing techniques [7]. The amounts of cyclohexanone, β-ocimene, styrene, and α-terpinene were considerably greater in the hydroponic plants when comparing the individual components across treatments. Phytochemicals found in aquaponic *H. odoratissimum* were much higher than those found in hydroponic and field-collected *H. odoratissimum*, including alpha-phellandrene, o-ethyltoluene, tetradecane, α-terpineol, α-curcumene, and palustrol [7]. Matrose et al. [8] explored the effects of spatial variation in South African *H. odoratissimum* gathered from the Western Cape and Kwazulu-Natal provinces, two distinct agroclimatic sites in South Africa. In all samples, eighteen different chemical groups of secondary metabolites were found. The most comparatively abundant were discovered to be sesquiterpenoids. A total of eighteen distinct chemical categories of secondary metabolites were discovered in all samples. Sesquiterpenoids (31–40%) were found to be the class of compound chemicals with the highest relative abundance, followed by sesquiterpenes (9–40%) and alkanes (7–12%). Sesquiterpenes were the most abundant in the acetone extract of Kwazulu-Natal *H. odoratissimum.* The acetone extract of *H. odoratissimum* from Kwazulu-Natal had higher sesquiterpenes. In comparison to samples from Kwazulu-Natal province, samples from the Western Cape had greater relative abundances of viridiflorol (26% and 16.8%, respectively, for acetone and ethanol extracts) (*p* ≤ 0.05). The results of this study are in line with previous research that found that the methanolic extracts of *H. odoratissimum* from Kwazulu-Natal, South Africa, have a comparatively high abundance of sesquiterpenoids and sesquiterpenes. In a complex sample of ethanolic leaf and stem extracts of *H. odoratissimum*, volatile compounds were separated and tentatively identified by GC-MS analysis [34]. The *H. odoratissimum* extracts contained a variety of compounds, the most common of which was 6-hydroxy-4-methoxy-2,3-dimethyl-benzaldehyde (20.23%), followed by tau-cadinol (6.53%), tetradecane (5.97%), and hexadecane (5.08%). The primary components of *Helichrysum* species that are frequently found in South Africa were examined by Adewinogo et al. [27]. The primary constituents of the sixty-three distinct chemical components found in the essential oils of the *Helichrysum* species were hydrocarbons, oxygenated monoterpenes, and sesquiterpenes. The main constituents of *H. odoratissimum* were 1,8-cineole (17.44%), α-pinene, and α-curcumene (15.76%). Monoterpenes and sesquiterpenes were the most frequently detected in several investigations aimed at tentatively identifying non-volatile secondary metabolites from *H. odoratissimum*. Furthermore, GC-MS was understandably mostly used as the detection method.

#### 2.2.2. Non-Volatile Compounds from *H. odoratissimum*

The flowers of *H. odoratissimum* have been shown to contain several non-volatile compounds. The acetone extract of *H. odoratissimum* flowers was found to contain 30,4′,3,5-tetrahydroxy-7-methoxyflavone (388) [27]. Van Puyvelde et al. [6] isolated two flavonoids 3,5-dihydroxy-6,7,8-trimethoxyflavone and 3-*O*-methylquercetin (389), and one chalcone, namely helichrysetin (1-(2,4-dihyroxy-6-methoxyphenyl)-3-(4-hydroxyphenyl)-2-propen-1-one) (390), from the *H. odoratissimum* flowers. *H. odoratissimum* contained 4,5-dicaffeoylquinic acid (391), which was reported for the first time from the species [12]. Sinda et al. [56] isolated compounds from *H. odoratissimun* aerial parts, ethyl acetate and *n*-butanol fractions were purified using varying chromatographic methods to afford 5,8-dihydroxy-3,6,7-trimethoxyflavone (392), 5,4′-dihydroxy-7-methoxyflavanone (393), methylinositol (394), quercetin (395), quercetin-3-methoxy-7-*O-β-*_D_-glucopyranoside (396), 3-hydroxydihydrobenzofurane (397), 4,5-di-*p*-*trans*-coumaroylquinic acid (398), kaempferol (399), β-sitosterol-3-*O*-*β*-_D_-glucopyranoside (400), and oleanolic acid (401). The few compounds that have been isolated from *H. odoratissimum* were mainly from flavonoid and triterpenoid classes. Perhaps one of the great limitations on the work conducted on *H. odoratissimun* is that few studies have focused on isolation or identification of non-volatile compounds that are more polar compared to the extensive work done on non-polar constituents.

### 2.3. Pharmacological Activities of H. odoratissimum Extracts, Fractions, Essential Oils, and Isolated Compounds

Owing to *H. odoratissimum’s* diverse traditional uses in the treatment of infectious diseases, skin-related conditions, TB and related symptoms, inflammation-related conditions, amongst others, different pharmacological activities of *H. odoratissimum* extracts, volatile and non-volatile secondary metabolite compounds such as antimicrobial, antimycobacterial, antioxidant, anti-inflammatory, antidiabetic, antiproliferative, antityrosinase and in vivo studies were reported. This was to ascertain or validate the traditional use scientifically. A table summarizing the most important pharmacological activity studies on *H. odoratissimum* and future research opportunities is presented in Table 3.

#### 2.3.1. Antimicrobial Activity

Most of the research on *H. odoratissimum* focused on identifying its antimicrobial properties, with a special emphasis on its antibacterial, antifungal, and antimycobacterial properties. The antibacterial activity of hexane and methanol extracts from the aerial parts of *H. odoratissimum* against *Aggregatibacter actinomycetemcomitans*, *Porphyromonas gingivalis*, *Tannerella forsythia*, *Streptococcus mutans*, *Streptococcus sobrinus*, and *Lactobacillus acidophilus* was examined by Ocheng et al. [45]. With the least inhibitory concentration (MIC) values between 0.125 and 0.5 mg/mL, the hexane extract was the most potent. MIC values for the methanol extract against the tested microorganisms ranged from 0.5 to 1 mg/mL [45]. Nevertheless, given that the antibacterial activity of crude extracts is considered significant if the MIC is less than 100 μg/mL [57,58,59], the activity was not pharmacologically significant. Even using the recent classification of antimicrobial activity by Eloff [60], which suggested that MICs ranging from <0.02 mg/mL as outstanding activity, 0.021–0.04 mg/mL as excellent activity, 0.041–0.08 mg/mL as very good activity, 0.081–0.16 mg/mL as good activity, 0.161–0.32 mg/mL as average activity, and >0.32 mg/mL as weak activity, the antimicrobial activity was not significant. De Canha [53] evaluated the antimicrobial activity of *H. odoratissimum* against *Propionibacterium acne*, a bacterium linked to the skin condition acne, using *p*-Iodonitrotetrazolium violet (INT) as a bacterial growth indicator and discovered an outstanding MIC of <3.91 µg/mL. Furthermore, *H. odoratissimum* showed the lowest MIC of 7.81 μg/mL against *Cutibacterium acnes* (previously *Propionibacterium acnes*) when prestoblue was used as an indicator. This remarkable antibacterial activity may explain its historical use in the treatment of skin-related conditions, while more research is required in this area. To investigate the potential combination of antimicrobial interactions, extracts from *Helichrysum odoratissimum* and *Helichrysum kraussii* were mixed at the same concentration of 2 mg/mL in nine different ratios (9:1; 8:2; 7:3; 6:4; 5:5; 4:6; 3:7; 2:8; 1:9). *Helichrysum* spp. alone had MIC values of 7.81 μg/mL for *H. odoratissimum* and 125.00 μg/mL for *H. krausii*, respectively. The fractional inhibitory concentrations (Σ FIC) were calculated in the synergistic assay. A synergistic effect was indicated by the combination’s computed Σ FIC value of 0.42. In particular, the antibacterial activity against *C. acnes* was higher when 3.13 μg/mL of *H. odoratissimum* and 0.78 μg/mL of *H. kraussii* were combined than when either plant extract was used alone. It was interesting to note that *H. odoratissimum* and *H. kraussii* at a variable ratio of 8:2 were found to be synergistic against *C. acnes* by the synergistic antimicrobial assay. The potential of *H. odoratissimum* methanolic extract to target bacterial growth and pathogenic virulence factors linked to acne advancement was examined in a follow-up investigation [55]. The methanol extract showed significant antibacterial activity against *C. acnes* American Type Culture Transfer (ATCC 6919) with a MIC value of 7.81 μg/mL. Additionally, the extract showed excellent selectivity against *C. acnes* cell aggregation at sub-inhibitory concentrations, inhibiting the development of biofilms. Mature *C. acnes* biofilms were disrupted at a sub-inhibitory concentration of 3.91 μg/mL, while the MIC for tetracycline, the positive control, was 0.78 μg/mL. The methanol extract showed a bactericidal effect at 250 μg/mL, according to the minimum bactericidal concentration (MBC) activity. Erythromycin, a macrolide antibiotic, was found to be less effective when combined with the methanol extract. When methanol and benzoyl peroxide (BPO) were mixed, the general trend was that less BPO was needed to exert an antibacterial effect at lower methanol extract (sub-MIC) concentrations. This is because the MIC of BPO alone was 31.25 μg/mL, whereas the MIC in combination with methanol extract and BPO was 5.47 μg/mL, 6.25 μg/mL, and 7.03 μg/mL [53].

Sinda et al. [56] evaluated the antibacterial activity of *H. odoratissimun* leaf extracts, fractions, and compounds against a range of bacteria and fungi. The ethanol extract, *n*-butanol, and ethyl acetate fractions were analyzed in addition to the eight isolated compounds. The ethyl acetate fraction was the most effective, with minimum inhibitory concentrations (MIC) of 16 µg/mL for *Pseudomonas aeruginosa* (ATCC 74117) and 32 µg/mL for *Candida albicans* (ATCC 9028). With a MIC of 64 µg/mL, the *n*-butanol fraction also demonstrated significant antibacterial activity against *C. albicans* (ATCC 9028). This was noteworthy as the polar fractions in this investigation were very active, yet the species is known to contain primarily volatile secondary metabolites, which are usually non-polar. With MIC values of 64 µg/mL, 64 µg/mL, and 32 µg/mL, respectively, 5,8-dihydroxy-3,6,7-trimethoxyflavone (392), 5,4´-dihydroxy-7-methoxyflavanone (393), and quercetin (394) demonstrated moderate antibacterial activity against *C. albicans*. Methylinositol (395) exhibited a minimum inhibitory concentration (MIC) of 128 µg/mL, but quercetin-3-methoxy-7-*O-β-*d-glucopyranoside (396) had the lowest MIC of only 256 µg/mL against *E. coli* (ATCC51299) and *C. albicans* (ATCC 9028). The minimum inhibitory concentration (MIC) of 3-hydroxydihydrobenzofurane (397) and 4,5-di-p-trans-coumaroylquinic acid (398) against *C. albicans* (ATCC 9028) was 256 µg/mL. According to the literature, compounds with MIC values of 10 μg/mL or less are thought to have significant pharmacological activity [58]. None of the identified compounds had antibacterial activities below the 10 μg/mL threshold. Much of the antimicrobial activity that was tested for *H. odoratissimum* extracts appeared to have been inspired by the study by Mathekga and Meyer [61], in which acetone extracts of *H. odoratissimum* were tested for antibacterial activities against ten bacteria using the agar diffusion method. *H. odoratissimum* extracts significantly reduced the growth of Gram-positive bacteria, such as *Bacillus cereus*, *B. pumilus*, *B. subtilis*, *Micrococcus kristinae*, and *Staphylococcus aureus*, as well as Gram-negative bacteria, such as *Enterobacter cloacae,* at concentrations between 0.01 and 1.0 mg/mL. However, it should be noted that the agar diffusion method was used, which has several shortcomings, particularly when dealing with medium-polar or non-polar extracts [62]. This may help explain why most of the subsequent work was conducted using the broth dilution strategy instead of the agar diffusion method. The antibacterial and antifungal properties of *H. odoratissimun* leaf and stem extracts extracted with chloroform: methanol (1:1) was determined against *B. cereus* ATCC 11778, *S. aureus* ATCC 12600, *Staphylococcus epidermidis* ATCC 2223, *Klebsiella pneumoniae* NCTC 9633, *Pseudomonas aeruginosa* ATCC 9027, and a yeast (*Cryptococcus neoformans* ATCC 90112). With an MIC value of 20 µg/mL against *S. aureus* and 30 µg/mL against *B. cereus*, *H. odoratissimum* demonstrated strong antibacterial activity. *H. odoratissimum* extracts showed modest antibacterial activity against *K. pneumoniae* and *S. epidermidis*, with MIC values of just 2 mg/mL and 4 mg/mL, respectively [62]. Using the broth microdilution method, the antibacterial activity of *H. odoratissimum*, which was shown to be high in 1,8-cineole (17.44%), and α-pinene (15.76%) in essential oils, was assessed against three skin pathogenic bacteria: *S. aureus*, *P. aeruginosa*, and *E. coli* [27]. The results showed that *H. odoratissimum* had low MIC values of 12.8 mg/mL against every tested bacterium. The antibacterial activity of *H. odoratissimum* methanol extracts against *E. coli*, *Proteus mirabilis*, *Staphylococcus saprophyticus*, *Enterococcus faecalis*, *Moraxella catarrhalis*, and *Streptococcus agalactiae* was evaluated [12]. Several extracts showed no activity, and the average MICs of the methanol extracts of the various samples varied from 0.60 mg/mL for *S. agalactiae* to 3.8 mg/mL for *P. mirabilis*.

The antifungal activity and secondary metabolite levels of *H. odoratissimum* extracts grown in hydroponic, aquaponic, and field systems against *Fusarium oxysporum* were compared by Zantanta et al. [7]. The ethanolic extracts of *H. odoratissimum* were fungistatic against *F. oxysporum*, according to the MIC results. The ethanol extracts of plants grown in the aquaponic system had a MIC value of only 0.37 mg/mL, followed by hydroponics with a MIC value of 0.56 mg/mL, and three field-collected plants produced the least activity of all the treatments tested, with a MIC value of 0.75 mg/mL. The study concluded that *H. odoratissimum* grown aquaponically showed the strongest antifungal efficacy, while the positive control, which employed the synthetic fungicide Dithane, had an MIC value of 0.75 mg/mL [7]. The antibacterial properties of *H. odoratissimum* essential oil against a few bacterial pathogens were identified by Lawal et al. [19]. According to the results, the *H. odoratissimum* oil had MIC values of 1.3 mg/mL against the three strains: *S. aureus* (ATCC 3983), *P. vulgaris* (CSIR 0030), and *S. faecalis* (ATCC 29212). These values were higher than the 100 µg/mL threshold for crude extract antimicrobial activity tests. Another study conducted in Uganda examined the antibacterial properties of *H. odoratissimum* against *Aggregatibacter actinomycetemcomitans*, *Porphyromonas gingivalis*, *Tannerella forsythia*, *Streptococcus sobrinus*, and *Lactobacillus acidophilus* using agar well-diffusion and agar dilution. With a minimum inhibitory concentration (MIC) of 0.125 mg/mL against *P. gingivalis*, *S. sobrinus*, and *L. acidophilus*, the hexane extract from the aerial portion of *H. odoratissimum* was the most effective [45]. Matrose et al. [8] investigated the antifungal activity of *H. odoratissimum* material collected from the Western Cape Province and the samples from Kwazulu-Natal Province against *Botrytis cinerea*. All extracts of *H. odoratissimum* provided considerable inhibition against *B. cinerea* in comparison to the controls. Furthermore, the ethanol extracts of *H. odoratissimum* were more efficacious than the acetone extracts against *B. cinerea* (*p* < 0.05) [8]. The isolated 3-O-Methylquercetin (389) was found to have noteworthy activity against the Gram-positive bacterium *Staphylococcus aureus* (MIC = 6.25 mg/mL) and the yeast *Candida albicans* (MIC = 12.5 mg/mL) [6].

Given that *H. odoratissimum* is traditionally used to treat tuberculosis and its associated symptoms in South Africa and Lesotho, it was somewhat of a surprise that there were very few studies on the antimycobacterial properties of this plant in the literature. One of those studies was an investigation of the antimycobacterial activity of the *H. odoratissimum* whole-plant extract against the pathogenic strain, *Mycobacterium tuberculosis* [19]. The study revealed that the extract had MICs ranging from 300 to 500 μg/mL against *M. tuberculosis*. Another study conducted by Seaman et al. [63] also indicated that the acetone extracts of *H. odoratissimum* inhibited *M. smegmatis* and *M. aurum* with MIC values ranging from 0.3 mg/mL to 2.0 mg/mL. Lall and Meyer [28] screened the whole *H. odoratissimum* plant for antimycobacterial activity against drug-resistant and drug-sensitive strains of *M. tuberculosis* H37Rv by the agar plate method. Acetone and water were used as extractants. *H. odoratissimum* acetone of the whole plant had an MIC of 0.5 mg/mL against the H37Rv strain of *Mycobacterium tuberculosis*, while the aqueous extract was not active at the highest concentration (5.0 mg/mL) tested. Considering that the species is abundant in volatile compounds, it will be worth investigating the antimycobacterial activity of the less polar extracts. Furthermore, other parts of the *H. odoratissimum* should be considered for future studies. In conclusion, although more in vivo research is required, *H. odoratissimum* extracts showed intriguing antimicrobial properties against the tested bacteria, mycobacteria, and fungi, supporting the traditional uses of this species.

#### 2.3.2. Antioxidant Activity

In a study by De Canha [55], *H. odoratissimum* had the highest DPPH radical scavenging activity with an IC_50_ of 3.86 ± 0.24 μg/mL. The results could not be compared with previously recorded data as the extracts were prepared from the flowers. The radical scavenging capacity of the extracts/lipophilic fractions of *H. odoratissimum* was determined using DPPH free radical inhibition, hydrophilic oxygen radical absorbance capacity (H-ORAC) assays and lipophilic oxygen radical absorbance capacity (L-ORAC) assays [34]. The antioxidant activity was 5.13 ± 0.07 μg/mL on DPPH, 2542 ± 34 μmol on H-ORAC and 3648 ± 72 μg/mL μmol on extracts. Twilley et al. [36] determined the antioxidant activity of *H. odoratissimum* ethanol leaf and stem extracts collected in Venda, Limpopo Province, South Africa using the 2,2-diphenyl-1-picrylhydrazyl (DPPH). The extracts had a radical scavenging activity assay with IC_50_ of 5.13 µg/mL, which were comparable to the positive control, ascorbic acid which had IC_50_ of 1.98 µg/mL. The antioxidant activity of the *Helichrysum* essential oils was evaluated by four in vitro antioxidant capacity assays: DPPH, ABTS, FRAP and ORAC assays [27]. In the FRAP assay, *H. odoratissimum* essential oils had the highest value of 3026.6 (6.1%) μmol AAE/L. In the DPPH assay, the essential oils were found to possess a very low percentage of radical scavenging activities (% RSA), with 4.09 ± 0.95 (2 mg/mL), 1.27 ± 0.43 (1 mg/mL), and −0.57 ± 0.03 (0.5 mg/mL). In the FRAP and ORAC assays, *H*. *odoratissimum* essential oils were found to exhibit the highest antioxidant capacities at 2 mg/mL with 3026.6 ± 184.6 µmol AAE/L and 6624.8 ± 10.8 µmol TE/L, respectively.

Free radical scavenging activity of the *H. odoratissimum* extracts was determined using the DPPH assay [8]. The antioxidant capacity of *H. odoratissimum* extracts obtained from Western Cape (WC) and Kwazulu-Natal was strongly dependent on the extraction solvents. The extracts made from 70% acetone from KZN had the maximum free radical scavenging activity of *H. odoratissimum* (14,205 ± 432 μmol TE g^−1^) when compared to the other extracts (*p* < 0.05). The ethanol extract from WC showed the lowest radical scavenging activity (9281 ± 704 μmol TE g^−1^) (*p* < 0.05), followed by acetone extracts from WC (13,251 ± 701 μmol TE g^−1^). The highest free radical scavenging activity of *H. odoratissimum* was observed for the extracts obtained with 70% acetone from KZN (14,205 ± 432 μmol TE g^−1^) compared to the other extracts (*p* < 0.05). This was followed by acetone extracts from WC (13,251 ± 701 μmol TE g^−1^), while the ethanol extract from WC had the least (9281 ± 704 μmol TE g^−1^) radical scavenging activity (*p* < 0.05). Zantanta et al. [7] investigated antioxidant activity of leaves of *H. odoratissimum* using 2,2-diphenyl-1-picrylhydrazyl (DPPH), ′2,2-Azino-bis(3-ethylbenzothiazoline-6-sulfonic acid) (ABTS), and ferric reducing antioxidant power (FRAP) free radical scavenging activities. The FRAP analysis of leaves of *H. odoratissimum* grown using different cultivation methods revealed no significant effect (DF = 2; *X*^2^ = 2.69; *p* = 0.14) of treatment on the antioxidant capacity of the plant extracts from the three cultivation techniques (aquaponic, hydroponic, and field-collected *H. odoratissimum*). Nevertheless, hydroponically grown *H. odoratissimum* had superior activity in the FRAP bioassay, with IC_50_ values of 3078.55 ± 355.44 µmol AAE/g compared to aquaponically grown 2350.46 ± 200.2196.50 ± 284.01 µmol AAE/g and plants taken in the field had 18 µmol AAE/g. On the DPPH assay, the leaf extracts from hydroponically grown plants exhibited higher antioxidant activity; nonetheless, there was no significant difference in the DPPH levels (mol TE/g) across the three cultivation methods (DF = 2; X^2^ = 0.91; *p* = 0.4). Similarly, the hydroponically grown *H. odoratissimum* produced a considerably better ABTS activity (µmol TE/g) than the aquaponic and field-collected *H. odoratissimum* (DF = 2; X^2^ = 8.44; *p* = 0.01). Hydroponic *H. odoratissimum* plants exhibited the highest antioxidant activity overall. The DPPH, ABTS and CUPRAC free radical scavenging activity of the whole extract of *H. odoratissimum* was also determined [64]. *H. odoratissimum* exhibited concentration-dependent increases in CUPRAC and FRAP, as well as DPPH and ABTS+ free radical scavenging activity. DPPH and ABTS^+^ levels were considerably (*p* < 0.001) higher at dosages 125–2000 µg/mL than at the lowest dose (7.8 µg/mL). Additionally, we observed a significant (*p* < 0.001) improvement in the plant extract’s CUPRAC at dosages of 500–2000 µg/mL as opposed to 7.8 µg/mL. *H. odoratissimum* and ascorbic acid both had dose-dependent lipid peroxidation inhibitory activity that was linked with DPPH, ABTS+, CUPRAC, and FRAP values. The proportion of lipid peroxidation inhibition did not differ significantly (*p* > 0.001) between the ascorbic and *H. odoratissimum* groups. In conclusion, the *H. odoratissimum* extracts demonstrated the expected radical scavenging action on the DPPH, ABTS, FRAP, and ORAC assays.

#### 2.3.3. Antiproliferative Activity

The sulforhodamine B (SRB) assay was used to measure the in vitro cytotoxicity of *H. odoratissimum* leaves and stems extracted with chloroform: methanol (1:1) against transformed human kidney epithelial (Graham) cells, breast adenocarcinoma (MCF-7), and glioblastoma (SF-268) cells at a concentration of 0.1 mg/mL [26]. Transformed human kidney epithelial cells (Graham cells, acquired from Dr. R Van Zyl, University of the Witwatersrand), MCF-7 breast adenocarcinoma, and SF-268 glioblastoma cells (obtained from the National Cancer Institute (NCI), USA) were used. A non-cancerous cell line was represented by the Graham cells. *H. odoratissimum* had 7.4% ± 0.7 cell viability against MCF-7, 17.5% ± 0.4 viability for Graham cells, and 48.2% ± 1.4 for SF-268 cells. Cytotoxicity of *H. odoratissimum* extracts on human monocyte cells (CRL 1593.2) (U937 cells) was determined and had an IC_50_ of 21.54 ± 0.56 μg/mL [52]. The U937 cell line (CRL1593.2) for the study was supplied by Highveld Biological (Pty) Ltd. (Modderfontein, Johannesburg, South Africa). The essential oil of *H. odoratissimum* also showed significant cytotoxicity against brine shrimp, with an LC_50_ of 31.62 µg/mL [19]. The anti-proliferative effect of *H. odoratissimum* methanol extracts was determined on human keratinocyte cells to determine non-lethal concentrations of the extract that could be investigated in subsequent hyper-keratinization and anti-inflammatory cell-based assays [55]. Murine macrophages (RAW264.7) were acquired from Merck SA (Pty) Ltd. (Sandton, Johannesburg, South Africa), and human keratinocytes (HaCaT) were supplied by Dr. Lester Davids of the Department of Human Biology, University of Cape Town. Methanol extracts from *H. odoratissimum* were reported to have an IC_50_ on HaCaT viability of 167.00 ± 27.95 μg/mL. In toxicity evaluation, by brine shrimp lethality bioassay, the essential oils had LC_50_ values lower than 1000 µg/mL considered bioactive. The cytotoxicity of the *H. odoratissimum* extract was investigated on the U937 cells to determine IC_50_ values [65]. *H. odoratissimum* had an IC_50_ of 20.3 ± 3.1 μg/mL. The next phase was then to determine the morphological haematoxylin and eosin staining was used to analyse the morphological characteristics of the A431 cells (Purchased from European Collection of Cell Cultures) after exposure to 15 µg/mL (IC_50_), 30 µg/mL (2IC_50_) and 7.5 µg/mL (½IC_50_) of *H. odoratissimum* and to determine what mechanism of cell death was taking place. The cytotoxicity of the *H. odoratissimum* extract was investigated on the U937 cells to determine IC_50_ values [65,66]. *H. odoratissimum* had an IC_50_ of 20.3 ± 3.1 μg/mL.

The next step involved analyzing the morphological features of the A431 cells following exposure to 15 µg/mL (IC_50_), 30 µg/mL (2IC_50_), and 7.5 µg/mL (½IC_50_) of *H. odoratissimum* using hematoxylin and eosin staining to identify the mechanism of cell death. After being exposed to the extract at concentrations of 15 µg/mL and 30 µg/mL, cells began to exhibit abnormal cell growth and symptoms of cell death. A condensed nucleus and cell debris with low cell viability were found at 30 µg/mL, while apoptotic body formation and cell shrinkage were reported at 15 µg/mL. Cells exposed to 7.5 µg/mL showed usual signs of cell death, including a damaged nucleus. Actinomycin D-treated cells showed signs of a compressed and damaged nucleus, substantial cell death, and total loss of viability. The cytotoxicity of the ethanol extract from the leaves was determined using the XTT (Sodium 3′-[1-(phenyl amino-carbonyl)-3,4-tetrazolium]-bis-[4-methoxy-6-nitro] benzene sulfonic acid hydrate) colorimetric assay, which measures the reduction in viable cells in the presence of the crude extract.

The extract showed strong cytotoxic activity against A431 cells with an IC_50_ of 15.5 ± 0.15 µg/mL. Furthermore, skin cancer cells were cytotoxically affected by the extracts. The extracts demonstrated moderate to strong cytotoxic activity when tested on non-cancerous cell lines, such as Chang liver cells, human embryonic kidney cells (Hek293), and mouse melanocyte cells (816F10), with IC_50_ values of 57.43 µg/mL, 37.1 ± 4.8 µg/mL, and 25.43 ± 0.55 µg/mL, respectively. The antiproliferative effects of the extracts on human dermal fibroblast cells were evaluated using the PrestoBlue cell viability reagent, which is predicated on viable cells’ ability to convert resazurin to resorufin [34]. The IC_50_ value of actinomycin D, which was utilized as the positive control to induce toxicity in the cells, was 0.022 ± 0.002 mg/mL. The IC_50_ value for *H. odoratissimum* was 90.62 ± 0.21 mg/mL, indicating that the two extracts performed similarly. The antiproliferative activity of ethanol extracts from *H. odoratissimum* leaves and stems was evaluated against human hepatocytes (HepG2), human fetal lung fibroblast (MRC-5), human small cell lung carcinoma (SHP-77), and non-small cell lung cancer (A549) [19]. The non-small cell lung cancer (A549), human small cell lung carcinoma (SHP-77), human hepatocytes (HepG2), murine macrophages (RAW 264.7), and human fetal lung fibroblast (MRC-5) were supplied by Separations Scientific (Pty) Ltd. (Johannesburg, South Africa). *H. odoratissimum* ethanol extracts had an IC_50_ of 83.43 ± 1.60 µg/mL against A549, 49.46 ± 0.48 µg/mL against SHP-77, 23.61 ± 1.06 µg/mL against HepG2, and 50.71 ± 2.27 µg/mL against MRC-5 cells. The antiproliferative effect of the *H. odoratissimum* ethanolic extract was evaluated against Beas-2B cells, another non-cancer line, and its antiproliferative activity against A549 cells was confirmed using the sulforhodamine-b (SRB) assay. The IC_50_ values, which measured 73.15 ± 3.63 µg/mL against A549 and 30.26 ± 6.14 µg/mL against Beas-2B cells, were similar to those discovered in the Prestoblue assay, with a selectivity index value of 0.4. To ascertain the type of cell death induced, the morphology of A549 cells was assessed in relation to the ethanolic extract of *H. odoratissimum*. The ethanolic extract of *H. odoratissimum* caused apoptosis at a concentration of 50 µg/mL and was shown by the development of apoptotic bodies and blebbing of the cell membrane. HepG2 cells were treated with an ethanolic extract of *H. odoratissimum* at a concentration of 50 µg/mL, and changes in their morphological features were assessed. Despite a decrease in cell death, the HepG2 cells treated with the ethanolic extract of *H. odoratissimum* had compacted chromatin and other morphological indicators of apoptosis [29].

Deeh et al. [66] recently carried out the cytotoxicity study of the aqueous extract of the whole *H. odoratissimum* using cell viability, hemolysis, and HET-CAM irritation assay. HaCaT cells used for the study were obtained from the Korean Cell Line Bank (KCLB, Seoul, ROK). Low to moderate doses of the plant extract, ranging from 7.8 μg/mL to 250 μg/mL, were found to enhance cell viability after treatment of HaCat cells (normal human keratinocytes) exposed to different concentrations of *H. odoratissimum* over 24 h. Additionally, *H. odoratissimum* was examined for its ability to protect HaCat cells from H_2_O_2_-induced cytotoxicity. Interestingly, all *H. odoratissimum* concentrations significantly (*p* < 0.001) increased the vitality of HaCaT cells in comparison to the H_2_O_2_ group, and this was explained by this species’ high antioxidant activity. This was attributed to the strong antioxidant activity of this species. The study also discovered that H_2_O_2_ adversely altered the morphology of HaCat cells after treatment. When compared to the control, it was evident that the H_2_O_2_-treated cells had irregular shapes, were smaller, and were longer. There were also a lot of apoptotic bodies. Interestingly, *H. odoratissimum* improved these aberrant traits. *H. odoratissimum* was shown to be hemolytic at doses of 1000 μg/mL to 2000 μg/mL and non-hemolytic at dosages of 7.8 μg/mL to 500 μg/mL in a hemolysis assay employing red blood cells. In general, *H. odoratissimum* did not produce cytotoxicity in red blood cells at doses 7.8 μg/mL to 500 μg/mL or on the hen’s egg-chorioallantoic membrane at all doses tested. However, an acute and chronic toxicity evaluation of *H. odoratissimum* in animal models to validate in vitro cytotoxicity is recommended.

#### 2.3.4. Anti-Inflammatory Activity

The 5-lipoxygenase inhibitory activity and major compounds identified in the oils of *H. odoratissimum* [67] were reported. All oils tested exhibited promising 5-lipoxygenase inhibitory activity with IC_50_ values between 35 ppm and 75 ppm. The oil of *H. odoratissimum* displayed the most promising 5-lipoxygenase inhibitory activity with an IC_50_ value of 35.90 ppm. Essential oils such as β-caryophyllene and limonene, which are also constituents from *H. odoratissimum,* have previously been shown to display strong 5-lipoxygenase inhibitory activity. The anti-inflammatory activity of *H. odoratissimum* was used to determine the levels of cytokine production (IL-8 and IL-12) ELISA kit [68]. *H. odoratissimum* enhanced the production of IL-8 compared to the production of IL-12 in the medium control from 5 µg/mL to 15 µg/mL. However, as the concentration of the extract increased, the production of IL-12 decreased, which could be due to the lowered viability of the cells. The production of IL-8 followed a similar pattern to that of IL-12, with a higher concentration of *H. odoratissimum* being associated with a lower production of IL-8. When compared to the medium control, the IL-8 was suppressed at all extract doses. Since DMSO was used to prepare the stock quantities of the extracts, it may potentially account for the inhibition observed in the extract. DMSO exhibited the strongest suppression of IL-8. The effects of *H. odoratissimum* methanol extracts, together with *C. acnes* stimulation, were used to assess the anti-inflammatory activity of *H. odoratissimum* methanol extracts in response to *C. acnes*-induced cytokine production specifically [55]. IL-1α increased from a basal value of 298.10 ± 20.88 µg/mL to 374.69 ± 10.17 µg/mL due to *C. acnes*. Additionally, 100 μg/mL *H. odoratissimum* methanol extracts significantly decreased IL-1α protein levels to 333.18 ± 10.92 µg/mL in cells treated with *C. acnes*. The anti-inflammatory activity of methanol extracts in response to *C. acnes*-induced cytokine production was particularly assessed using the effects of *H. odoratissimum* methanol extracts and caused *C. acnes* [55]. It was discovered that the levels of IL-6 and IL-8 proteins were induced within measurable bounds, and this was documented. It was subsequently determined that reduction of IL-6 or IL-8 was not caused by cytotoxicity against HaCaT cells for methanol extracts because more than 70% of HaCaT cells were viable following treatment. IL-8 protein levels were reduced by 48.31%, from 561.58 ± 14.85 to 290.24 ± 21.02 µg/mL, when methanol extracts were evaluated at a MIC of 7.81 μg/mL. Methanol extracts demonstrated a dosage response in their COX-2 inhibitory action, with an IC_50_ of 22.87 ± 6.48 μg/mL. Linoleic acid, which has been shown to inhibit the COX enzyme in the past, was identified as the possible cause of the activity. The findings also matched research on other *Helichrysum* species. Methanol extracts showed an IC_50_ of 214.90 ± 13.27 μg/mL for nitric oxide (NO) [55]. The NO results suggested that methanol extracts of *H. odoratissimum* were more efficacious than those of other *Helichrysum* species. The inhibition of NO was then determined through the concentration of nitrite (NO^2−^) and compared with lipopolysaccharides-induced RAW264.7 cells. The *H. odoratissimum* methanol extracts had significant inhibition of NO at 7.81 μg/mL, 15.625 μg/mL, and 31.25 μg/mL. Furthermore, the concentration of nitrite was reduced from 19.46 μM in the LPS-stimulated cells to 14.86 μM, 12.31 μM, and 11.60 μM, respectively. The gene expression of several inflammatory genes related to acne, including COX-2 and iNOS, were investigated in LPS-induced RAW264.7 cells, treated with *H. odoratissimum* methanol extract, with the β-actin gene as the reference (housekeeping) gene [55]. The methanol extracts exhibited inhibition of COX-2 expression, even though there was a negligible difference between the COX-2 expression levels in the methanol extract treatments when compared to those observed in LPS-induced expression. Flavonoid compounds previously isolated from the methanolic flower extract of *H. odoratissimum* may explain the anti-inflammatory activity of the methanol extract used in the study. It was recommended that future studies could focus on flavonoid-rich extracts or fractions of *H. odoratissimum* for anti-inflammatory activity.

*H. odoratissimum* leaves and stems ethanol extracts were investigated for their anti-inflammatory potential against RAW 264.7 murine macrophage cells, Cyclooxygenase-2 (COX-2) and 5-Lipoxygenase (5-LOX) enzymes, and for their inhibitory potential against NQO1 [29]. The ethanolic extract of *H. odoratissimum* had an IC_50_ value of 60.15 ± 1.98 µg/mL against murine macrophages (RAW 264.7) cells. This study demonstrated that at 10 µg/mL, the *H. odoratissimum* ethanolic extract had inhibitory activity of 76.14 ± 9.52% for COX-2 and an IC_50_ value of 7.94 ± 3.84 µg/mL for COX-2, while the inhibitory activity for 5-LOX was 87.17 ± 5.32% and an IC_50_ value of 2.08 ± 1.35 µg/mL for 5-LOX. The positive controls ibuprofen and zileuton had IC_50_ values of 0.85 ± 0.14 µg/mL (COX-2) and 0.06 ± 0.05 µg/mL (5-LOX) [29]. Esmear et al. [29] measured the levels of NQO1 in A549 cells to determine whether the ethanolic extract of *H. odoratissimum* decreased the expression of NRF-2. There was no noticeable distinction between the untreated control and the 0.1% DMSO control. At 125 and 250 mg/mL, the ethanolic extract drastically reduced NQO1 expression in comparison to the positive control brusatol (500 nM). Evaluating the effect of *H. odoratissimum* aqueous extracts on wound healing and confirming the traditional usage of this species for wound healing was of extreme interest to Deeh et al. [64]. The effects of the drugs on wound healing are frequently tested using HaCat cells as an experimental model. HaCat cells are widely used as an experimental model to test the effect of drugs on wound healing. In all groups, the percentage of wound closure was time dependent. After 12, 24, and 48 h of treatment, we discovered that *H. odoratissimum* significantly (*p* < 0.05–0.01) enhanced keratinocyte migration as compared to the control group. For instance, after 12 h, 24 h, and 48 h of treatment with *H. odoratissimum* (at a dose of 125 μg/mL), the wound closure ratio was 29%, 83%, and 100%. At the end of the incubation period (48 h), *H. odoratissimum* at a dose of 125 μg/mL was the most active, since 100% of wound closure was recorded. Various phytocompounds found in *H. odoratissimum* could be responsible for the wound healing effect of this plant. These results support the use of *Helichrysum* species as wound healing enhancers in traditional medicine, subject to confirmation through clinical trials. In general, *H. odoratissimum* showed interesting anti-inflammatory activities via different mechanisms such as lipoxygenase, cyclooxygenase, nitric oxide, and cytokine.

#### 2.3.5. Antiparasitic Activity

In one of the very few studies to investigate the parasitic activity of the aerial parts of *H. odoratissimum*, the chemical composition, repellent, and oviposition deterrent effects of essential oils extracted from *H. odoratissimum* were evaluated against *Aedes aegypti*, *Anopheles gambiae*, and *Culex quinquefasciatus*. When tested at 33.3 μg/cm^2^, *H. odoratissimum* was an effective repellent against *Ae. aegypti* with 51% repellency, but it was less effective against *An. gambiae* with 49% repellency. Interestingly, *H. odoratissimum* exhibited 100% repellency against *Cx. quinquefasciatus* at 33.3 μg/cm^2^ [30]. The contact, repellent and fumigation effects of essential oils from *Helichrysum odoratissimum* were investigated against maize weevil, *Sitophilus zeamais* (Motschulsky) (Colepotera: Curculionidae). The level of repellency caused by the essential oils of *H. odoratissimum* was 49.4%. The study revealed that the essential oils of *H. odoratissimum* had weak contact and fumigation effects against *S. zeamais* [46].

#### 2.3.6. Mutagenicity

Twilley et al. [34] tested *H. odoratissimum* ethanol and dichloromethane extracts for potential mutagenicity/genotoxicity using the Ames test with *Salmonella typhimurium* strains TA98 and TA100, which detect frame-shift mutations and base-pair substitutions, respectively. The Ames test identifies a genotoxic/mutagenic sample if the number of revertant colonies formed on the plate containing the test sample is twice the number of colonies formed on the DMSO/sterile water only control plate. The number of revertant colonies formed on *Salmonella typhimurium* TA98 revertant colonies was 6.67 ± 4.04 (5 mg/mL), 50.00 ± 6.08 (0.5 mg/mL), and 61.00 ± 9.89 (0.05 mg/mL), and the number of *Salmonella typhimurium* TA100 revertant colonies was 119.33 ± 10.02 (5 mg/mL), 129.33 ± 8.39 (0.5 mg/mL), 127.67 ± 20.50 (0.05 mg/mL). Both extracts showed statistically different (*p* < 0.01) results from the positive control at each of the tested concentrations. Furthermore, the plates containing each of the extracts did not form more than twice the number of revertant colonies as in the dimethyl sulfoxide (DMSO) control plate; therefore, the extracts had no mutagenic effect on the *Salmonella* strains. Both extracts had results that were statistically different (*p* < 0.01) from the positive control at all tested concentrations. Furthermore, the plates containing each extract did not yield more than twice as many revertant colonies as the DMSO control plate, indicating that the extracts had no mutagenic effect on the *Salmonella* strains.

#### 2.3.7. Antidiabetic Activity

Ngagi et al. [32] carried out the hypoglycaemic potential of the *H. odoratissimum* aqueous leaf extracts in alloxan-induced diabetic mice. Three dose ranges were used: 50 mg/kgbwt, 100 mg/kgbwt and 150 mg/kgbwt. The results were then measured and observed at different time intervals. In the first hour, none of the tested dosages of the aqueous leaf extracts from *H. odoratissimum* significantly lowered blood glucose levels. In the 2nd hour, the three dose levels lowered the blood sugar levels by 30%, 54%, and 54%, respectively. At this hour, the aqueous extracts lowered blood glucose levels to normal, but not as effectively as insulin (*p* < 0.05; *p* < 0.05). Interestingly, glucose decreased by the same percentage with dosages of 100 mg/kgbwt and 150 mg/kgbwt. The percentage decrease in blood glucose levels for the three dosing levels was 26%, 62%, and 70% in the third hour. As in the second hour, the blood glucose level dropped by the dose range of 50 mg/kg and 100 mg/kg body weight. However, the dose range of 150 mg/kg body weight reduced blood sugar levels to below normal and was just as effective as insulin. The aqueous leaf extracts maintained their dose-dependent hypoglycemic action in the fourth hour, increasing by 38%, 67%, and 75%, respectively. At this stage, blood glucose levels were brought below normal by the 100 mg/kg and 150 mg/kg body weight dose ranges, and the 150 mg/kg body weight dose had a hypoglycemic potential similar to that of insulin. The aqueous leaf extracts of *H. odoratissimum* showed a non-dose dependent response. This observation could suggest that the aqueous leaf extracts could have been actively absorbed in the cell system. Animals administered leaf extracts from *H. odoratissimum* showed mild perivascular inflammation in their kidneys, but the renal cells were unaffected. The spleen tissue cells remained intact, apart from a minor decline in lymphocytes. There was mild perihepatitis, but hepatocytes were unharmed, and the liver showed no signs of illness. There was no cardiac muscle damage. The absence of noticeable pathological abnormalities in mice given high dosages of *H. odoratissimum* aqueous leaf extracts is encouraging evidence that the extract is a safe herbal alternative for treating diabetes mellitus. It is possible that drug-induced reactions are the only cause of the inflammation seen at the injection site. Thus, the in vitro anti-diabetic potential of *H. odoratissimum* aqueous extracts of the whole plant was studied by estimating the α-amylase and α-glucosidase activities [64]. It is well known that the inhibition of these enzymes decreases glucose absorption at the level of the intestine. In the present study, the authors found that *H. odoratissimum* exhibited potent antidiabetic activity in vitro by inhibiting the activity of α-amylase and α-glucosidase in a dose-dependent manner from 2000 µg/mL to 7.8 µg/mL concentrations tested in both studies. However, acarbose, used as a reference drug, showed the highest activity. Future studies on the in vitro antidiabetic effect of *H. odoratissimum* should be compared with other commonly used antidiabetic drugs such as thiazolidinediones, biguanides, and sulfonylureas.

#### 2.3.8. Anti-Tyrosinase Activity

Adewinogo [27] determined the in vitro tyrosinase inhibitory activities of the essential oils using mushroom tyrosinase. The absorbance of L-3,4-dihydroxyphenylalanine (L-DOPA) was monitored at λ490 nm using L-tyrosine as a substrate. The Kojic acid positive control and essential oil samples were evaluated at 200 μg/mL and 50 μg/mL. The findings indicated that at 200 μg/mL and 50 μg/mL, the essential oils had inhibitory activities of 63.30 ± 2.35–51.53 ± 10.30% and 28.62 ± 0.30–19.13 ± 0.81%, respectively. Deeh et al. [64] assessed the effects of *H. odoratissimum* aqueous extracts from the whole plant on L-DOPA auto-oxidation and tyrosinase inhibition using L-DOPA and L-tyrosine as substrates. Based on the data, we observed that *H. odoratissimum* suppressed L-DOPA auto-oxidation and tyrosinase in a dose-dependent way. Additionally, *H. odoratissimum* (dosage 62.5–2000 μg/mL) had stronger tyrosinase inhibition and L-DOPA auto-oxidation values than the ascorbic acid used as a positive control. Additionally, we observed that when L-DOPA was utilized as the substrate, the percentage of tyrosinase inhibition was higher than the values obtained when tyrosine was used. Since L-tyrosine and L-DOPA are tyrosinase’s substrate and cofactor, respectively, *H. odoratissimum* primarily functions as a tyrosinase cofactor. The strong antioxidant properties of *H. odoratissimum* aqueous extract, along with its high levels of total phenols and flavonoids, may be responsible for its anti-tyrosinase effect.

#### 2.3.9. Clinical Trials on *H. odoratissimum*

In accordance with the Declaration of Helsinki and the Guidelines for Good Laboratory Practice in the Conduct of Clinical Trials in Human Participation in South Africa, the irritancy potential of the *H. odoratissimum* ethanol extracts was investigated [34]. The Research and Ethics Committee of Sefako Makgatho Health Sciences University granted permission to conduct the study (MREC/H/48/2014: CR; as renewed), and each participant provided written informed consent. Five of the twenty adult female participants (ages 18 to 65) who were enlisted for the study had sensitive skin. Exactly 19 of the 20 volunteers who were recruited finished the study; the 20th volunteer was unable to do so because of unrelated circumstances. There were no adverse events that were reported during the study. Following the application of the extracts, erythema was assessed visually and using a chromameter at 0, 24, 48, 72, and 96 h. While there was no statistically significant difference between the extracts and the deionized water control, the irritancy potential of the positive control was significantly higher than that of the extracts and the deionized water control. The irritancy values were classified according to the following: mean + SD > 1.5 = sample is an irritant; mean + SD ≤ 1.5 and >negative control (deionized water) = sample has low irritancy potential, and mean + SD ≤ negative control = sample is a non-irritant [67]. According to the classification, *H. odoratissimum* was classified as a low irritant; it should be noted that the irritancy testing was conducted using the neat, reconstituted plant extracts (6.0 mg/mL) and not using the sunscreen formulations with the incorporated extracts. The neat testing was performed to evaluate the highest potential of irritancy that could be displayed when using the extracts. The detailed visual mean scores (+ SD) showed that the positive control was a high irritant after 48 h of application; 1.35 (24 h), 1.75 (48 h), 2.03 (72 h) and 2.05 (96 h), whereas deionized water showed no irritancy at each of the time intervals; 0.69 (24 h), 0.85 (48 h), 0.45 (72 h) and 0.52 (96 h). *H. odoratissimum* showed slightly higher erythema scores; 1.10 (24 h), 1.28 (48 h), 1.02 (72 h) and 0.96 (96 h). The chromameter results were used to verify the visual scores of erythema. The positive control was confirmed to be highly irritant at each of the time intervals; 2.88 (24 h), 3.48 (48 h), 3.94 (72 h) and 3.49 (96 h), whereas deionized water was much lower; 1.48 (24 h), 1.76 (48 h), 1.28 (72 h) and 0.90 (96 h). The reading of *H. odoratissimum* was higher than deionized water but still lower than that of the positive control; 1.81 (24 h), 2.10 (48 h), 1.30 (72 h) and 0.88 (96 h).

#### 2.3.10. In Silico Studies

In computational research, molecular docking is a useful technique for figuring out the interactions and binding energies between a drug and its target.

Deeh et al. [64] investigated the molecular docking of selected phyto-constituents identified in *H. odoratissimum* against tyrosinase (PDB ID: 2Y9X) and L-DOPA (PDB ID: 6ON3) to analyze the ligand-protein interaction. p-menthone, eucalyptol, 2-cyanoacetamide, 2(5 h)-furanone, 5-methyl- and Pulegone had interesting affinities. The binding pattern between mushroom tyrosinase or L-DOPA and each secondary metabolite varied depending on the ligand structure. P-menthone relates to four alkyl/pi-alkyl bonds and several van der Waals interactions with the protein’s active site. Additionally, it showed a significant tyrosinase binding affinity (−8.76 kcal/mol). Eucalyptol, on the other hand, was more stable because it contained several van der Waals and Alkyl/Pi-Alkyl interactions in addition to two typical hydrogen bonds. Two conventional hydrogen bonds connected 2-cyanoacetamide to the mushroom tyrosinase binding site, whereas one conventional hydrogen bond connected 2-(5 h)-furanone, 5-methyl-. Pulegone had a high affinity of −8.39 kcal/mol, which can be explained by its interaction with tyrosinase via a single conventional hydrogen bond with the Asn-2029 residue, as well as strong van der Waals and alkyl/pi-alkyl interactions. With a binding affinity of −10.07 kcal/mol, p-menthone connected with the active pocket of L-DOPA by two traditional hydrogen bonds with the residues Arg-640 and Thr-637. The binding affinities of 2(5 h)-furanone, 5-methyl, and 2-cyanoacetamide with L-DOPA, on the other hand, were −6.43 kcal/mol and −5.69 kcal/mol, respectively, through a single conventional hydrogen bond. Although eucalyptol, pulegone, and L-DOPA did not form traditional hydrogen bonds, they did show strong Alkyl/Pi-Alkyl and van der Waals interactions with the enzyme. Overall, we discovered that p-menthone had the highest binding affinity with tyrosinase (−8.76 kcal/mol) and L-DOPA (−10.07 kcal/mol) out of all the chosen phytochemical compounds. These results clearly demonstrated the ability of all the chosen bioactive substances from *H. odoratissimum* to suppress the activities of tyrosinase and L-DOPA.

The molecular interaction between the chemicals found in *H. odoratissimum* and α-amylase is shown to involve a single typical hydrogen bond between the His-305 residue of α-amylase and p-menthone or pulegone [64]. The active site of α-amylase did not exhibit any traditional hydrogen bonds with eucalyptol or 2(5 h)-furanone or 5-methyl; nevertheless, they did exhibit strong hydrophobic π-alkyl and Van der Waals interactions, respectively. On the other hand, 2-cyanoacetamide interacted with Arg-124, Asp-138 and Asn-137 of the enzyme via three conventional hydrogen bonds, confirming its best stability. In comparison to acarbose (−7.54 kcal/mol), which was used as a reference inhibitor, the binding affinities of p-menthone (−8.49 kcal/mol), eucalyptol (−7.90 kcal/mol), and pulegone (−8.59 kcal/mol) were higher. However, acarbose showed the highest stability with α-amylase because it had four conventional hydrogen bonds with the residues Thr-376, Asp-375, Asp-456, and Arg-392. Acarbose is widely used as a reference drug for in vivo, in vitro, and in silico antidiabetic assays. The selected phytochemical compounds used a similar binding method to α-glucosidase’s binding pocket as α-amylase. For example, p-menthone (−9.26 kcal/mol), eucalyptol (−8.61 kcal/mol), and pulegone (−9.72 kcal/mol) display greater binding affinities with α-glucosidase than acarbose (−8.54 kcal/mol). Eucalyptol and p-menthone interacted with the binding site of α-glucosidase via one and two conventional hydrogen bonds, respectively, while 2-cyanoacetamide showed three conventional hydrogen bonds with the enzyme. A stronger binding affinity between the ligand and the protein is indicated by higher negative values during docking analysis, which suggests that the phytocompounds are more effective. Therefore, pulegone had the highest affinity for α-glucosidase at −9.72 kcal/mol. These in silico and in vitro data provide a strong basis for studying *H. odoratissimun* in in vivo animal models.

#### 2.3.11. Other Pharmacological Activities

Despite extensive work done on *H. odoratissimum*, perhaps the limitations are that very few studies focused on in vivo studies to validate some of the interesting pharmacological studies. In one of the few in vivo studies, Watcho et al. [31] investigated the effects of aqueous extracts (AE) and methanolic extracts (ME) of *H. odoratissimum* for reducing cyclophosphamide-induced reproductive toxicity in male rats. In comparison to the control group, the cyclophosphamide-treated group exhibited a significant decrease (*p* < 0.001) in body and seminal vesicle weights, testosterone levels, sperm count, sperm motility, and sperm viability, but an increase (*p* < 0.001) in sperm morphological abnormalities and testicular structure alterations. It is noteworthy that these adverse effects of cyclophosphamide were mitigated using *H. odoratissimum* extracts. Both aqueous extracts (AEs) and methanolic extracts (MEs) and all doses of *H. odoratissimum* significantly increased the sperm count (*p* < 0.001), sperm motility (AE, 50 mg/kg, *p* < 0.05; ME, 50 and 100 mg/kg, *p* < 0.05) and sperm viability (AE, 50 mg/kg, *p* < 0.001; ME, 50 and 100 mg/kg, *p* < 0.001) compared to the cyclophosphamide group. Additionally, *H. odoratissimum* reduced histological changes to the testes and increased intratesticular and plasmatic testosterone levels. De Canha et al. [55] determined the hyaluronidase (HYAL) inhibitory activity of *H. odoratissimum* methanolic extracts using the turbidometric assay. The methanolic extract showed dose-dependent suppression of HYAL activity, with an IC_50_ of 145.45 ± 6.22 μg/mL. The inhibitory activity of the methanol extract was comparable with previously published data on bacterial hyaluronidase inhibitors, which showed that flavonoids are reported to have an inhibitory effect on hyaluronidase assay. Considering that flavonoids and diterpenes have previously been isolated from *H. odoratissimum,* it is not surprising that methanol extracts showed inhibition of this enzyme. Since there are currently no known treatments for acne that target hyaluronidase inhibition, more research into identifying the constituents of *H. odoratissimum* methanol extracts as hyaluronidase inhibitors is necessary. The methanol extracts exhibited an IC_50_ of 157.50 ± 6.85 μg/mL against lipase activity. Tetracycline showed low levels of lipase inhibition even at the highest concentration tested [55]. With regard to stability testing of formulation, a minor increase in pH between cycle 0 and cycle 1 was noted, after which the pH remained constant [34]. The average pH value obtained over all the cycle testing for the formulation was determined as 6.81 ± 0.17 for *H. odoratissimum,* which showed no significant variation in the pH values over the complete cycle test period. The in vitro UVA sun protection factor (SPF) was further measured pre-irradiation and post-irradiation to determine the photostability of the sunscreens [34]. There was no significant difference in each of the tested parameters post-irradiation, indicating the photostability of the sunscreen formulations. *H. odoratissimum* extracts showed a lower decrease in in vitro SPF compared to the base sunscreen formulation post-irradiation. The sunscreen with the addition of the extracts showed a reduction of 0.2 in SPF for *H. odoratissimum*, whereas the base sunscreen showed a decrease of 0.38 post-irradiation, indicating that the extracts contributed to an incremental photoprotective effect.

Human external cutaneous ageing is mostly caused by solar UV radiation, which can lead to a variety of dermatological issues, including skin cancer. The absorbance of diluted hydroalcoholic essential oil solutions (0.1% *v*/*v*) at 290–320 nm at 5 nm intervals was measured to assess the SPF absorbance of the *Helichrysum* essential oils [27]. The study demonstrated that the essential oils of *H. odoratissimum* had an SPF of 0.309. *H. odoratissimum* essential oils may not be significant for sunscreen compositions because the SPF values were below the acceptable range. Esmear et al. [29] determined the effect of the *H. odoratissimum* ethanolic extracts on angiogenesis using the ex ovo yolk sac membrane (YSM) assay. The *H. odoratissimum* ethanolic extract at 18.5 µg/egg had a 31.65 ± 12.80% inhibition of blood vessel formation, while the positive control, combretastatin A4 (CA4), had a 45.12 ± 8.49% inhibition at 10 nmol/egg. The results showed that there was no statistical difference between the activity of CA4 and the *H. odoratissimum* ethanolic extract; however, the extracts had a lower amount of newly formed blood vessels compared to CA4.

**Table 3:** Some pharmacological activities studies on *H. odoratissimum* to explore its traditional uses and future research opportunities.

Traditional Uses of *H. odoratissimum*	Validated Pharmacological Activities	Research Gaps and Potential Future Studies
**Wound dressing and burns.**	The results of the wound healing potential of *H. odoratissimum* in in vivo studies validated the use of this species [64].	Though the extract has potential as a wound healing enhancer in traditional medicine in an in vivo study, more studies are still recommended, especially clinical trials.
**An ingredient to formulate a skincare product (sunscreen).**	The irritability potential of the *H. odoratissimum* ethanol extracts was investigated in clinical trials in South Africa [34]. The extract has some irritants, though classified as low irritants.	The future clinical trials should consider combinational studies with other species that are used to treat skin conditions or other products that are used to treat skin conditions.
**Teething syndrome**	The n-butanol fraction also had antifungal activity against *C. albicans* with a MIC of 64 µg/mL [56].	Future studies should consider using clinical isolate strains to determine oral candidiasis. Other studies can also focus on the activity of the extracts against other oral pathogens.
**Decoction is used as a treatment for pimples and other skin-related conditions.**	Antimicrobial activity against *C. acnes*, a bacterium linked to the skin condition of acne and had MIC of <3.91 µg/mL [50]. The n-butanol fraction also had antifungal activity against *C. albicans* with a MIC of 64 µg/mL [56]. *H. odoratissimum* suppressed L-DOPA auto-oxidation and tyrosinase in a dose-dependent way [64].	Isolation of the bioactive compounds responsible for the good activity against *Propionibacterium acnes* should be explored. Antifungal activity of the active fractions should be further investigated in in vivo studies in instances where it is not feasible to isolate a large quantity of the active compounds. More studies should also focus on other pharmacological assays that are used to determine skin-related conditions.
**Treatment of tuberculosis.**	Whole-plant extract had MICs ranging from 300 to 500 μg/mL against the *M. tuberculosis* pathogenic strain [17]. Acetone extract had an MIC of 0.5 mg/mL against H37Rv strain of *M. tuberculosis* [28].	The antimycobacterial activity of the extract should focus on both non-polar and polar extracts. Future studies should consider combined studies with TB drugs that are already in use.
**Mixed with *Olea europaea* and *Zantedeschia albomaculata* for backache.**	Anti-inflammatory activity to determine the levels of cytokine production (IL-8 and IL-12), Nitric oxide, Cyclooxygenase-2 and 5-lipoxygenase [65].	There is an obvious lack of in vivo studies, though various inflammation mechanisms have been investigated in vitro.

## 3. Materials and Methods

### 3.1. Literature Search Approach

Several internet databases, including Science Direct, Scopus, Google Scholar, Web of Science, SciFinder, Wiley Online, SpringerLink, and PubMed, provided the information presented in this study. Phytochemistry, essential oils, in silico studies, traditional uses, pharmacological activity, biological activity, antimicrobial activity, antibacterial, antifungal, antimycobacterial, antioxidant, anti-inflammatory, antidiabetic, antiproliferative activity, antiparasitic, cytotoxicity, toxicity, clinical trials, ex vivo, and in vivo were among the keywords associated with *H. odoratissimum*.

### 3.2. Study Selection and Compilation

Publications were collected from the different databases until 31 December 2025. All recorded publications were then screened based on their titles, abstracts, and keywords to determine their inclusion or exclusion from this review. After preliminary screening, a total of 91 articles were chosen based on the relevance of their titles, evaluating their relevance for the review by reading the abstract, downloading and reading the full article. At least 45 articles contained relevant information based on the inclusion criteria and scope of the review. The following criteria were used:

Inclusion Criteria

- Access to English full-text articles or articles in any other language with the option to have them translated to English.
- Publications with relevant information published prior to 31 December 2025.
- Published peer-reviewed articles on ethnopharmacology, pharmacological activity, traditional uses, phytochemistry, secondary metabolites, in silico studies, clinical trials, in vivo studies, and essential oils of *H. odoratissimum*.

Exclusion Criteria

- Articles that were not available for translation into English or that were not published in English were not included.
- Articles that had information about the plant, such as ecology-related work or other information outside of the scope of the present review, were omitted
- Letters, encyclopedia, manuals, and guidelines were excluded.

## 4. Conclusions and Future Perspectives

*H. odoratissimum* is widely distributed in southern Africa and other parts of Africa and the world. It is widely used as an ethnomedicine to treat or prevent infectious diseases, TB, inflammation-related conditions, skin-related disorders, wound healing and other conditions. Studies on the essential oil composition and other volatile compounds of *H. odoratissimum* showed monoterpenoids and sesquiterpenoids as mostly the main compounds. Several studies also reported the isolation of the non-volatile compounds, which were mainly flavonoids and terpenes. The species has been reported to have pharmacological activities such as antimicrobial, antimycobacterial, antioxidant activity, antidiabetic, antiproliferative, anti-inflammatory activity, and antityrosinase activities.

The most important study on *H. odoratissimum* was the safety trial involving human participants in South Africa to assess in vivo irritancy potential. It was interesting to note that there were limited toxicity studies reported for *H. odoratissimum*. The isolation study of *H. odoratissimun* led to the isolation and characterization of ten non-volatile compounds. The ethanol extract, ethyl acetate and butanol fractions, and some of the isolated compounds were evaluated for their antimicrobial activity and showed significant activity against *C. albicans*. When it is not feasible to isolate a high percentage of the bioactive compounds, the antifungal activity of the active fractions should be further examined in in vivo investigations. Clinical isolate strains of oral candidiasis should be used in future studies. Other studies can also focus on the antifungal activity of the extracts against other oral pathogens. The EtOAc fraction showed significant activity against the bacterial strain *P. aeruginosa*. The ethyl acetate fraction could be chosen for the development of a Phyto drug against microbial diseases in in vivo studies. The results may explain the use of *H. odoratissimun* in traditional medicine against infectious diseases.

The investigation of the synergistic antimicrobial activity of the extracts, the combination of *H. odoratissimum* and *Helichrysum kraussii* showed better antimicrobial activity than either of the plant extracts acting alone against *C. acnes,* which further supports the use of *H. odoratissimum* in combination with other plant species. Furthermore, pharmacological activities should explore the activities of combined extracts. The studies conducted provided scientific validation for the traditional use of *H. odoratissimum* as a possible treatment for acne, based on direct antimicrobial effects as well as the inhibition of key targets in the pathogenic processes associated with the opportunistic pathogen, *C. acnes,* in the progression of this skin disorder. The study identified the potent antimicrobial activity of the methanolic extract of *H. odoratissimum* against *C. acnes* and the anti-biofilm activity of this extract, particularly affecting the initial step of bacterial adhesion. Other in vitro and/or in vivo pharmacological assays that are used to identify skin-related diseases should also be the subject of future research. Isolation of the bioactive compounds from *H. odoratissimum* responsible for the good activity against *C. acnes* should be explored.

The ethanolic extract demonstrated antiproliferative activity against both the A549 and SHP-77 lung cancer cell lines and induced apoptotic cell death in the A549 cells. The studies conducted also identified the anti-inflammatory potential of *H. odoratissimum* inhibiting inflammatory cytokine IL-8, COX-2, and NO. The mechanism of action for NO inhibition was through the inhibition of iNOS gene expression. The anti-inflammatory effect of the ethanolic extract indicated that the extract inhibited both COX-2 and 5-LOX enzymes. The ethanolic extract inhibited the production of IL-8 and promoted the production of IL-12 in U937 cells. Although in vitro anti-inflammatory activity *H. odoratissimum* has been studied through several inflammatory mechanisms, there is a clear lack of in vivo research in this regard. *H. odoratissimum* improved the viability of HaCat cells and protected the cells against H_2_O_2_ cytotoxicity. Moreover, *H. odoratissimum* did not cause any cytotoxicity in red blood cells or on hen’s egg-chorioallantoic membrane at the concentrations tested, at all doses, but further in vivo studies are required to confirm the safety of this plant.

In vitro studies revealed the anti-tyrosinase, anti-α-amylase, anti-α-glucosidase, and wound healing activities of *H. odoratissimum*, which were also supported by the in silico findings. Future studies on the in vitro antidiabetic effect of *H. odoratissimum* should compare with other commonly used antidiabetic drugs such as thiazolidinediones, biguanides, and sulfonylureas. Perhaps the most important results of *H. odoratissimum* extracts are that they could be considered a valuable addition to sunscreen formulations, as they displayed an increased stabilizing and photoprotective effect, showed significant antioxidant activity, were non-mutagenic, and could be considered relatively safe for cosmetic use as evaluated by the irritancy test on clinical trials in South Africa. However, further research on the clinical and scientific aspects is needed to justify some of their other medicinal uses that are yet to be validated in vitro and in vivo. Furthermore, combinational studies with other species used to treat skin disorders or other products used to treat skin conditions should be considered for consideration for future clinical trials.

## Figures and Tables

**Table 1:** Medicinal uses and corresponding parts of *Helichrysum odoratissimum*.

Medicinal Part	Traditional Uses	References
Leaves	Wound dressing and burns. An ingredient to formulate a skincare product (sunscreen). Sotho people use it to fumigate huts. Teething syndrome Leaf ashes are ingested to relieve vomiting. Tea from leaves is used to treat colic and stomachache. Extracts or sap from the leaves and twigs of the plant may be used as eye drops to treat conjunctivitis. Sap from the leaves is used as eye drops to treat conjunctivitis. Infusion of leaves is used to treat symptoms associated with fever. Leaves are burnt, and the smoke is inhaled, which acts as a sedative or to treat insomnia. Leaves are burned as incense. Decoction is used as a treatment for pimples. Treatment of tuberculosis.	[1,13,15,17,18,34,37,38,39,40,43,44,47,50,51]
Roots	Coughs and colds. Orally administered as a colonic cleanser.	[1]
Twigs	Extracts from twigs are used as eye drops to treat conjunctivitis	[1]
Whole Plant	Decoction is used to treat abdominal pains, heartburn, wounds, female sterility, menstrual pain and/or eczema. Decoction is used as an ointment for the treatment of pimples. Burnt as a fumigant for flu, coughs and colds. Mixed with *Olea europaea* and *Zantedeschia albomaculata* for backache. Treatment of tuberculosis and related symptoms such as colds/coughs.	[1,6,15,16,17,18,48,49]
Aerial parts	Used to treat dehydration	[1]
Stem	Stem is burnt, and the smoke is inhaled, which acts as a sedative or treats insomnia	[1,15,37,38,39]

**Table 2:** Tentatively identified volatile compounds from *Helichrysum odoratissimum*.

	RI	Compounds	MW (g/mol)	MF	% Area	Plant Part	Detection Method	References
1	893	Decane	142.29	C_6_H_9_NO_4_	0.26	Leaves, stem	GC-MS	[7,34]
2	934–940	α-Pinene	136.234	C_10_H_16_	4.11–47.1	Flowers, leaves	GC-MS, IR, MS	[5,7,19,21,27,46,50]
3	929	Nonadecane	268.518	C_19_H_4_0	3.25	Leaves, stem	GC-MS	[7,34]
4	845–955	Camphene	136	C_10_H_16_	0.12–3.06	Flowers, leaves, stem	GC-MS, IR, MS	[5,8,34]
5	-	4-Methyl-octane	128.26	C_9_H_20_	0.60–2.69	Leaves	GC-MS	[5,7,8,21,27,46]
6	970–981	*β*-Pinene	136.23	C_10_H_16_	0.1–6.25	Leaves, flowers, stem	GC-MS, IR, MS	[5,7,8,21,27,46]
7	884	Undecane	156.31	C_11_H_24_	1.05, 1.91	Leaves, stem	GC-MS	[7,34,55]
8	1006	*α*-Phellandrene	136.23	C_10_H_16_	Traces	Leaves, flowers	GC-MS	[5,7]
9	981–993	Myrcene	136.23	C_10_H_16_	0.3–2.20	Leaves, flowers	GC-MS, IR, MS	[5,7,21,27,46]
10	-	*β*-Phellandrene	136.24	C_10_H_16_	0.33–0.51	Leaves	GC-MS	[7]
11	1025–1036	1,8-Cineole	154.249	C_10_H_18_O	0.13–17.44	Leaves, flowers, stem	GC-MS, IR, MS	[5,7,8,46]
12	-	*O*-Ethyltoluene	120.19	C_9_H_12_	0.18–2.13	Leaves	GC-MS	[5,7]
13	1028–1051	*β*-Ocimene	136.24	C_10_H_16_	0.3–2.7	Leaves, flowers	GC-MS, IR, MS,	[5,7,21,27]
14	1045–1062	γ-Terpinene	136.234	C_10_H_16_	0.3–2.54	Leaves, flowers	GC-MS, IR, MS	[5,7,27,46]
15	-	Styrene	104.15	C_8_H_8_	0.22–0.67	Leaves	GC-MS	[7]
16	-	ρ-Cymene	134.21	C_10_H_14_	5.18–14.76	Leaves	GC-MS	[7]
17	-	1,2,3-Trimethylbenzene	120.19	C_9_H_12_	0.01, 5.91	Leaves	GC-MS	[7]
18	-	*α*-Fenchene	136.23	C_10_H_16_	0.22–1.38	Leaves	GC-MS	[7]
19	1082–1097	*α*-Terpinolene	136.23	C_10_H_16_	0.15–2.44	Leaves, flowers, stem	GC-MS	[5,7,8,19,21,46]
20	-	Cyclohexanone	98.15	C_6_H_10_O	1.07–4	Leaves	GC-MS	[7]
21	-	2,6,6-Trimethylcyclohexanone	140.22	C_9_H_16_O	0.01–0.16	Leaves	GC-MS	[7]
22	-	3-Hexenyl acetate	142.20	C_8_H_14_O_2_	1.47–3.07	Leaves	GC-MS	[7]
23	-	2-Heptenal	112.17	C_7_H_12_O	1.52–3.19	Leaves	GC-MS	[7]
24	1348	6-Methyl-5-hepten-2-one	126.20	C_8_H_14_O	1.07–2.31	Leaves	GC-MS	[7]
25	1186	Allo-ocimene	136.23	C_10_H_16_	0.41–3.33	Leaves	GC-MS	[7]
26	1386	Octenyl acetate	170.25	C_10_H_18_O_2_	1.1–4.98	Leaves	GC-MS	[7]
27	-	3-Hexenol	100.16	C_6_H_12_O	0.21–0.62	Leaves	GC-MS	[7]
28	-	4-Methyl-1,5-heptadiene	110.2	C_8_H_14_	0.04–0.28	Leaves	GC-MS	[7]
29	1393	3-Octanol	130.23	C_8_H_18_O	Traces	Leaves	GC-MS	[7,52]
30	-	3-Ethyl-o-xylene	134.22	C_10_H_14_	0.20–0.62	Leaves	GC-MS	[7]
31	934, 1659.8	Tetradecane	198	C_14_H_30_	5.97	Leaves, stem	GC-MS	[7,8,34]
32	1452	1-Octen-3-ol	128.22	C_8_H_16_O	0.1–14.15	Leaves	GC-MS	[7,52]
33	1482	*β*-Fenchyl acetate	196.29	C_12_H_20_O	0.1–13.77	Leaves, aerial parts	GC-MS	[7,52]
34	-	6-Methyl-5-hepten-2-ol	128.21	C_8_H_16_O	0.30–4.59	Leaves	GC-MS	[7]
35	-	2,5-Dimethyl-*p*-xylene	134.22	C_10_H_14_	0.51–4.67	Leaves	GC-MS	[7]
36	1373–1493	*α*-Ylangene	204.35	C_15_H_24_	Traces,0.4	Leaves	GC-MS	[7,8,21,52]
37	1409	Italicene	204.35	C_15_H_24_	2.93–23.43	Leaves	GC-MS, IR, MS	[7]
38	520, 660	Benzaldehyde, 6-hydroxy-4-methoxy-2,3-dimethyl-	180	C_10_H_12_O_3_	0.23–26.18	Leaves, stem	GC-MS	[7,34]
39	-	Allyl isopentanoate	142.20	C_8_H_14_O	5.24–12.70	Leaves	GC-MS	[7]
40	1472–1487	γ-Curcumene	204.35	C_15_H_24_	2.15–22.09	Leaves, stem	GC-MS, IR, MS	[7,8,27,46]
41	1084–1100	L-linalool	154.25	C_10_H_18_O	0.5–52.53	Leaves	GC-MS	[7,19,21,46]
42	1376, 1384	*α*-Copaene	204.35	C_15_H_24_	0.6–55.57	Leaves, flowers, stem	GC-MS	[5,7,8,19,21,50]
43	1072	*Cis*-Sabinene hydrate	154.25	C_10_H_18_O	0.01–1.08	Leaves, flowers	GC-MS	[5,7]
44	1508	*E,E-α*-Farnesene	204.35	C_15_H_24_	0.7–4.67	Leaves	GC-MS	[7,19]
45		Fenchol	154.25	C_10_H_18_O	1.40–2.28	Leaves	GC-MS	[7]
46	1400, 1429	*β*-caryophyllene	204	C_15_H_24_	0.6–15	Leaves, flowers, stem	GC-MS	[5,7,8,21,50]
47	1453.6	(+)-Aromadendrene	204.35	C_15_H_24_	0.3, 2.7	Leaves, stem	GC-MS	[7,8]
48	1340	δ-Elemene	204.35	C_15_H_24_	0.63, 0.27	Flowers, leaves	GC-MS	[5,7]
49	-	(-)-Isoledene	204.35	C_15_H_24_	1.50–2.35	Leaves	GC-MS	[7]
50	-	Ethyl-caprate	200.32	C_12_H_24_O_2_	1.05–2.23	Leaves	GC-MS	[7]
51	-	Pinocarveol	152.23	C_10_H_16_O	0.59–2.15	Leaves	GC-MS	[7]
52	1450–1461	*α*-Humulene	204.35	C_15_H_24_	0.13–6	Leaves, flowers	GC-MS, IR, MS	[5,7,19,21,27,50]
53	-	Linaly-propanoate	210.31	C_13_H_22_O_2_	7.35–100.91	Leaves	GC-MS	[7]
54	-	Acoradiene	204.35	C_15_H_24_	6.4–28.41	Leaves	GC-MS	[7]
55	-	1,8-Menthadien-4-ol	152.23	C_10_H_16_O	4.11–12.61	Leaves	GC-MS	[7]
56	1499	*β*-Himachalene	204.36	C_15_H_24_	0.32	Leaves, flowers	GC-MS	[5,7]
57	1176–1193	*α*-Terpineol	154.25	C_10_H_18_O	0.2–5.51	Leaves, flowers	GC-MS, IR, MS	[5,7,19,21,27]
58	1505	Ledene	204.35	C_15_H_24_	1.1–5.6	Leaves, stem	GC-MS	[7,8]
59	-	(+)-2-Carene	136.23	C_10_H_16_	369.88	Leaves	GC-MS	[5,7,21,55]
60	1495, 1493	Valencene	204.35	C_15_H_24_	0.88–3.23	Leaves, flowers, stem	GC-MS	[5,7,21,55]
61	1410	α-Gurjunene	204.37	C_15_H_24_	0.26–1.19	Leaves, flowers	GC-MS	[5,7]
62	1486.0	Eremophilene	204.36	C_15_H_24_	0.1–1.19	Leaves	GC-MS	[7,8]
63	1485, 1496	β-Selinene	204.35	C_15_H_24_	1.72–3.03	Leaves, flowers, stem	GC-MS	[5,7,8]
64	-	Neryl acetate	196.29	C_12_H_20_O_2_	27.44–45.58	Leaves	GC-MS	[7]
65	-	*α*-Bisabonele	264.4	C_17_H_28_O_2_	8.84–16.43	Leaves	GC-MS	[7]
66	-	*β*-Bisabonele	220.35	C_15_H_24_O	6.76–12.93	Leaves	GC-MS	[7]
67	-	*α*-Cedrene	204.35	C_15_H_24_	10.33–15.72	Leaves	GC-MS	[7]
68	-	7-Epi-*α*-Selinene	204.35	C_15_H_24_	17.24–25.47	Leaves	GC-MS	[7]
69	1500–1523	δ-Cadinene	204.36	C_15_H_24_	0.35–13.18	Leaves, flowers, stem	GC-MS, RI, MS	[5,7,8,19,21,27,46,50]
70	1475	α-Curcumene	202	C_15_H_22_	3.74–20.3	Leaves, stem	GC-MS	[7,21,55]
71	-	Ar-curcumene	202.33	C_15_H_22_	180.67	Leaves	GC-MS, RI, MS	[7,26]
72	1497.0	γ-Selinene	204.35	C_15_H_24_	0.4, 1.4	Leaves, stem	GC-MS	[7,8]
73	1532, 1541	α-Cadinene	204.35	C_15_H_24_	0.40–3.2	Leaves, flowers, stem	GC-MS	[5,7,8]
74	-	Nerol	154.25	C_10_H_18_O	0.33–0.91	Leaves	GC-MS	[7]
75	-	2-Phenylethyl acetate	164.20	C_10_H_12_O_2_	2.32–5.93	Leaves	GC-MS	[7]
76	-	Isogeraniol	154.25	C_10_H_18_O	0.42–2.26	Leaves	GC-MS	[7]
77	-	β-Damascenone	190.28	C_13_H_18_O	0.42–2.26	Leaves	GC-MS	[7]
78	-	1S-calamenene	202.34	C_15_H_22_	0.34–5.48	Leaves	GC-MS	[7]
79	1845, 1882	Carveol	152.23	C_10_H_16_O	0.1–0.2	Leaves	GC-MS	[52]
80	-	P-cymen-8-ol	150.22	C_10_H_14_O	0.2	Leaves	GC-MS	[7,52]
81	-	4-Phenyl-2-butanone	148.20	C_10_H_12_O	0.61–0.83	Leaves	GC-MS	[7]
82	-	Ethyl laurate	228.37	C_14_H_28_O_2_		Leaves	GC-MS	[7]
83	1452	*(E)*-Geranyl acetone	194.32	C_14_H_24_O	0.2–1.3	Leaves, stem	GC-MS	[7,8]
84	-	Ascaridole	168.23	C_10_H_16_O_2_	2.68–5.57	Leaves	GC-MS	[7]
85	-	Benzyl alcohol	108.14	C_7_H_8_O	0.72–1.42	Leaves	GC-MS	[7]
86	-	4-Ethyl-o-xylene	134.22	C_10_H_14_	2.08–3.21	Leaves	GC-MS	[7]
87	-	Ethyl-3-phenylpropionate	178.23	C_11_H_14_O_2_	2.15–3.24	Leaves	GC-MS	[7]
88	-	Phenylethyl alcohol	122.16	C_8_H_10_O	3.19–6.57	Leaves	GC-MS	[7]
89	1539, 1542	α-Calacorene	200.32	C_15_H_2_O	0.40	Flowers, leaves, stem	IR, MS, GC-MS	[5,7,8,27]
90	-	Palustrol	222.37	C_15_H_26_O	0.82–3.38	Leaves	GC-MS	[7]
91	1351	α-Cubebene	204.36	C_15_H_24_	0.6–3.47	Leaves, stem	GC-MS	[7,8]
92	1578–1583	Caryophyllene oxide	220.35	C_15_H_24_O	0.2–1.66	Leaves	IR, MS, GC-MS	[5,8,27,46]
93	-	(+)-Ledol	222.37	C_15_H_26_O	2.01–2.42	Leaves	GC-MS	[7]
94	-	*α*-Caryophyllene alcohol	222.37	C_15_H_26_O	0.71–0.89	Leaves	GC-MS	[7]
95	1621	Fonenol	220.35	C_15_H_24_O	13.65–15.36	Leaves	GC-MS	[7]
96	-	Longifolenaldehyde	220.35	C_15_H_24_O	11.63–13.88	Leaves	GC-MS	[7]
97	-	N-benzylidenecyclohexylamine	187.28	C_13_H_17_N	3.47–4.47	Leaves	GC-MS	[7]
98	-	Cyclooctanone	126.20	C_8_H_14_O	4.74–7.54	Leaves	GC-MS	[7,34]
99	-	Caryophyll-5-en-2-beta-οl	220.35	C_15_H_24_O	1.54–3.36	Leaves	GC-MS	[7]
100	2187	T-cadinol	222.37	C_15_H_26_O	0.1–0.2	Leaves	GC-MS	[7,52]
101	-	Eugenol	164.2	C_10_H_12_O	12.06–17.22	Leaves	GC-MS	[7]
102	1435.0	(+)-Calarene	204.35	C_15_H_24_	0.3, 4.8	Leaves, stem	GC-MS	[7,8]
103	1695	Eudesm-7(11)-en-4-ol (Juniper camphor)	222.37	C_15_H_26_O	0.17–1.98	Leaves, flowers	GC-MS	[5,7]
104	-	β-Cadinene	204.35	C_15_H_24_	1.39–1.55	Leaves	GC-MS	[7]
105	1488	Epi-bicyclosesquiphellandrene	204.35	C_15_H_24_	0.2–2.27	Leaves, stem	GC-MS	[7,8]
106	1301	Carvacrol	150.22	C_10_H_14_O	0.35	Leaves, flowers	GC-MS	[5,7]
107	-	α-Eudesmol	222.37	C_15_H_26_O	0.62–8.06	Leaves	GC-MS	[7]
108	-	β-eudesmol	222.37	C_15_H_26_O	2.22–2.48	Leaves	GC-MS	[7]
109	-	Decanoic acid	172.26	C_10_H_20_O_2_	2.37–4.03	Leaves	GC-MS	[7]
110	-	(-)-Phyllocladene	272.5	C_20_H_32_	6.46–9.58	Leaves	GC-MS	[7]
111	-	2,7-Dimethyl-1,6-octadiene	138.25	C_10_H_18_	5.83–11.11	Leaves	GC-MS	[7]
112	-	Xanthorrhizol	218.33	C_15_H_22_O	1.95–3.34	Leaves	GC-MS	[7]
113	972	β-Pinene	136.23	C_10_H_16_	51.6	Leaves	GC-MS	[21]
114	1022–1029	Limonene	136.24	C_10_H_16_	0.13–16.9	Leaves, stem	GC-MS	[5,8,19,21,46]
115	1382	β-Elemene	204.35	C_15_H_24_	0.4	Leaves	GC-MS	[19]
116	1423	α-Caryophyllene	204.35	C_15_H_24_	4.7	Leaves	GC-MS	[19]
117	1448–1463	(E)-β-Farnesene	204.35	C_15_H_24_	0.4–2.1	Leaves	GC-MS	[19,21]
118	1481	Germacrene D	204.35	C_15_H_24_	2.3	Leaves	GC-MS	[19]
119	1562	(*E*)-Nerolidol	222.37	C_15_H_26_O	1.1	Leaves	GC-MS	[19]
120	1603	Humulene epoxide II	220.35	C_15_H_24_O	0.4	Leaves	GC-MS	[19]
121	1749	Drimenol	222.37	C_15_H_26_O	0.7	Leaves	GC-MS	[19]
122	1017, 1019	α-Terpinene	136.23	C_10_H_16_	0.15–1.77	Leaves, flowers	GC-MS, IR, MS	[5,27]
123	1170–1186	Terpinen-4-ol	154.25	C_10_H_18_O	0.63–1.04	Leaves	GC-MS, IR, MS	[21,27,46]
124	14.22	(*E*)-Caryophyllene	204.35	C_15_H_24_	7.30	Leaves	IR, MS	[27]
125	1514	Sesquicineole	222.37	C_15_H_26_O	2.75	Leaves	IR, MS	[27]
126	968	Sabinene	136.23	C_10_H_16_	0.3–0.9	Leaves	GC-MS	[21]
127	1216	Bornyl formate	182.26	C_11_H_18_O	0.29, 0.16	Flowers, leaves	GC-MS	[5,21]
128	1405	Santalene	204.35	C_15_H_24_	0.6, 0.9	Leaves	GC-MS	[21]
129	1487	Germacrene-D	204.35	C_15_H_24_	2.6, 5.1	Leaves	GC-MS	[21]
130	1498	ε-Cadinene	204.35	C_15_H_24_	Traces	Leaves	GC-MS	[21]
131	1500, 1505, 1741	β-Bisabolene	204.35	C_15_H_24_	0.4–1.8	Leaves	GC-MS	[8,21,52]
132	1496.6–1513	trans-γ-Cadinene	204.36	C_15_H_24_	0.1–3.9	Leaves, aerial parts	GC-MS	[5,8,21,54]
133	1527–1533	trans-Cadina-1,4-diene (Cubebene)	204.35	C_15_H_24_	0.6–2.3	Flowers, leaves, stem	GC-MS	[5,8,21]
134	934	Tricyclene	136.23	C_10_H_16_	0.56	Flowers	GC-MS	[5]
135	1019	para-Methylanisole	122.16	C_8_H_10_O	1.29	Flowers	GC-MS	[5]
136	1026	O-Cymene	134.22	C_10_H_14_	0.11–0.77	Flowers, leaves	GC-MS	[5]
137	1104	Nonanal	142.24	C_9_H_18_O	Traces	Flowers, leaves	GC-MS	[5]
138	1114	1-Octen-3-yl acetate	170.25	C_10_H_18_O_2_	0.22–0.41	Flowers, leaves	GC-MS	[5]
139	1130	α-Campholenal	154.25	C_10_H_18_O	Traces	Flowers, leaves	GC-MS	[5]
140	1148, 1151	Camphor	152.23	C_10_H_16_O	0.34	Flowers, leaves	GC-MS	[5,27]
141	1169	Borneol	154.25	C_10_H_18_O	Traces	Leaves	GC-MS	[5]
142	1156	Isoborneol	154.25	C_10_H_18_O	0.33	Flowers	GC-MS	[5]
143	1165	*neo*-Menthol	156.26	C_10_H_20_O	Traces	Flowers	GC-MS	[5]
144	1180	4-Terpineol	154.25	C_10_H_18_O	0.20–0.52	Flowers, leaves	GC-MS	[5]
145	1206	Decanal	156.27	C_10_H_20_O	Traces	Flowers	GC-MS	[5]
146	1285	Isobornyl acetate	196.28	C_12_H_20_O_2_	2.93	Flowers	GC-MS	[5]
147	1285	Bornyl acetate	196.28	C_12_H_20_O_2_	Traces	Flowers, leaves	GC-MS	[5]
148	1300	4-Terpineol acetate	196.29	C_12_H_20_O	0.21	Flowers, leaves	GC-MS	[5]
149	1300	*trans*-Pinocarvyl acetate	194.27	C_12_H_18_O_2_	0.15, 0.11	Flowers, leaves	GC-MS	[5]
150	1327	Myrtenyl acetate	194.27	C_12_H_18_O_2_	0.15	Flowers, leaves	GC-MS	[5]
151	1371	Cyclosativene	204.35	C_15_H_24_	0.43	Flowers, leaves	GC-MS	[5]
152	1384	trans-Myrtanol acetate	196.29	C_12_H_20_O_2_	0.38	Flowers	GC-MS	[5]
153	1404	Longifolene	204.35	C_15_H_24_	Traces	Flowers, leaves	GC-MS	[5]
154	1429	β-Copaene	204.35	C_15_H_24_	Traces	Flowers	GC-MS	[5]
155	1437	α-trans-Bergamotene	204.35	C_15_H_24_	0.30	Flowers	GC-MS	[5]
156	1440, 1438	α-Guaiene	204.35	C_15_H_24_	0.11–4.77	Flowers, leaves, stem	GC-MS	[5,8]
157	1461	*allo*-Aromadendrene	204.35	C_15_H_24_	0.13, 3.63	Flowers, leaves	GC-MS	[5]
158	1475, 1478, 1707	β-Chamigrene	204.35	C_15_H_24_	0.1–0.42	Flowers, leaves, stem	GC-MS	[5,8,52]
159	1495	Viridiflorene	204.35	C_15_H_24_	0.43–2.51	Flowers, leaves	GC-MS	[5]
160	1450	α-Himachalene	204.35	C_15_H_24_	7.32, 5.32	Flowers, leaves	GC-MS	[5]
161	1476	γ-Himachalene	204.35	C_15_H_24_	1.37	Leaves	GC-MS	[5]
162	1480	α-Amorphene	204.35	C_15_H_24_	0.20, 0.12	Flowers, leaves	GC-MS	[5]
163	1503	Germacrene A	204.35	C_15_H_24_	11.83, 10.73	Flowers, leaves	GC-MS	[5]
164	1518	Cubebol	222.37	C_15_H_26_O	0.27	Flowers	GC-MS	[5]
165	1542	Selina-3,7(11)-diene	204.35	C_15_H_24_	1.90, 1.40	Flowers, leaves	GC-MS	[5,50]
166	1556	Germacrene B	204.35	C_15_H_24_	0.24	Flowers	GC-MS	[5]
167	1586	Gleenol	222.37	C_15_H_26_O	Traces	Leaves	GC-MS	[5]
168	1606	5,7-Di-epi-α-eudesmol	222.37	C_15_H_26_O	0.25, traces	Flowers, leaves	GC-MS	[5]
169	1623	10-epi-γ-Eudesmol	222.37	C_15_H_26_O	0.37, 0.10	Flowers, leaves	GC-MS	[5]
170	1630	1-epi-Cubenol	222.37	C_15_H_26_O	2.3, 1.04	Flowers, leaves	GC-MS	[5]
	1636	Caryophylla 4(14),8(15)-dien-5-ol	220.35	C_15_H_24_O	0.16	Leaves	GC-MS	[5]
171	1642	epi-α-Cadinol	222.37	C_15_H_26_O	0.52	Leaves	GC-MS	[5]
172	1654	Himachalol	222.37	C_15_H_26_O	1654	Flowers	GC-MS	[5]
173	1651	α-Muurolol	222.37	C_15_H_26_O	4.31, 0.30	Flowers, leaves	GC-MS	[5]
174	1654	α-Eudesmol	222.37	C_15_H_26_O	1.71	Leaves	GC-MS	[5]
175	1655	α-Cadinol	222.37	C_15_H_26_O	0.43	Leaves	GC-MS	[5]
176	1672	β-Bisabolol	222.37	C_15_H_26_O	0.11	Flowers	GC-MS	[5]
177	1666	Bulnesol	222.37	C_15_H_26_O	3.59, 2.23	Flowers, leaves	GC-MS	[5]
178	1667	Intermedeol	222.37	C_15_H_26_O	0.24	Flowers	GC-MS	[5]
179	1676	Cadalene	198.30	C_15_H_18_	0.83	Flowers	GC-MS	[5]
180	1929	Cembrene	272.47	C_20_H_32_	0.31	Leaves	GC-MS	[5]
181	1978	Bifloratriene	272.5	C_20_H_32_	11.58, 7.43	Flowers, leaves	GC-MS	[5]
192	1478	*α*-Bulnesene	204.35	C_15_H_24_	0.3–4.4	Whole plant	GC-MS	[8,46,50]
193	-	Phytol	296	C_20_H_40_O	1.6, 2.20	Leaves, stem, whole plant	GC-MS	[50,55]
194	-	19,19-Dimethyl-eicosa-8,11-dienoic acid	336.6	C_22_H_40_O_2_	3.8	Whole plant	GC-MS	[50]
195	-	Ethyl linolenate	306.5	C_20_H_34_O_2_	3.9	Whole plant	GC-MS	[50]
196	-	Methyl octadec-9-en-12-ynoate	292.5	C_19_H_32_O_2_	2.2	Whole plant	GC-MS	[50]
197	-	Myristic acid	228.37	C_14_H_28_O_2_	1.4	Whole plant	GC-MS	[50]
198	-	Palmitic acid	256	C_16_H_32_O_2_	27.1	Leaves, stem, whole plant	GC-MS	[50,55]
	846	2(5H)-Furanone, 5,5-dimethyl-	112	C_6_H_8_O_2_	0.53	Leaves, stem	GC-MS	[34]
199	878	2(3H)-Furanone, dihydro-5-methyl-	100	C_5_H_8_O_2_	0.53	Leaves, stem	GC-MS	[34]
200	893	N,N,O-Triacetylhydroxylamine	159	C_6_H_9_NO_4_	0.16	Leaves, stem	GC-MS	[34]
201	919	Eucalyptol	154	C_10_H_18_O	2.06	Leaves, stem	GC-MS	[34]
202	805	Pentanoic acid, 2-methyl-3-oxo-, ethyl ester	158	C_8_H_14_O_3_	2.70	Leaves, stem	GC-MS	[34]
203	844	2-Hexanone, 6-bromo-	178	C_6_H_11_BrO	0.30	Leaves, stem	GC-MS	[34]
	759	3-(5-Methylfuryl)-N-furamidopropionamide	262	C_13_H_14_N_2_O_4_	0.09	Leaves, stem	GC-MS	[34]
204	943	Benzoic acid, ethyl ester	150	C_9_H_10_O_2_	0.11	Leaves, stem	GC-MS	[34]
	856	Terpineol	154	C_10_H_18_O	0.50	Leaves, stem	GC-MS	[34]
205	910, 200.12	Dodecane	170	C_12_H_26_	4.3–1.4	Leaves, stem	GC-MS	[8,34]
206	866	3,5-Diamino-1,2,4-triazole	99	C_2_H_5_N_5_	0.23	Leaves, stem	GC-MS	[34]
207	894	Benzothiazole	135	C_7_H_5_NS	0.77, 2.18	Leaves, stem	GC-MS	[34]
208	745	Acetate, 4-hydroxy-3-methyl-2-butenyl-	144	C_7_H_12_O_3_	1.14	Leaves, stem	GC-MS	[34,55]
209	784	Cyclohexanol, 2,4-dimethyl-	128	C_8_H_16_O	1.35	Leaves, stem	GC-MS	[34]
210	730	Bicyclo[3.1.1]heptan-3-ol, 2,6,6-trimethyl-, [1R-(1à,2á,3á,5á)]-	154	C_10_H_18_O	0.66	Leaves, stem	GC-MS	[34]
211	719	Pseudosolasodine diacetate	499	C_31_H_49_NO_4_	1.68	Leaves, stem	GC-MS	[34]
	831	Hexanoic acid, anhydride	214	C_12_H_22_O_3_	0.58	Leaves, stem	GC-MS	[34]
212	819	2,5-Dimethyl-2-(2-tetrahydrofuryl)tetrahydrofuran	170	C_10_H_18_O_2_	0.99	Leaves, stem	GC-MS	[34]
213	865	à-Muurolene	204	C_15_H_24_	0.71	Leaves, stem	GC-MS	[34]
214	893	Naphthalene, 1,2,3,4,4a,5,6,8a-octahydro-7-methyl-4-methylene-1-(1-methylethyl)-, (1à,4aá,8aà)-	204	C_15_H_24_	3.85	Leaves, stem	GC-MS	[34]
215	833	Naphthalene, 1,2,3,5,6,8a-hexahydro-4,7-dimethyl-1-(1-methylethyl)-, (1S-cis)-	204	C_15_H_24_	2.74	Leaves, stem	GC-MS	[34]
216	756	Naphthalene, 1,2,4a,5,6,8a-hexahydro-4,7-dimethyl-1-(1-methylethyl)-, [1S-(1à,4áa,8aà)]-	204	C_15_H_24_	1.34	Leaves, stem	GC-MS	[34]
217	719	à-Calacorene	200	C_15_H_20_	1.34	Leaves, stem	GC-MS	[34]
218	882	p-Nitrophenyl hexanoate	237	C_12_H_15_NO_4_	0.78	Leaves, stem	GC-MS	[34]
219	932	Hexadecane	226	C_16_H_34_	5.08	Leaves, stem	GC-MS	[8,34]
220	876	tau-Cadinol	222	C_15_H_26_O	6.53	Leaves, stem	GC-MS	[34]
221	866	2-Naphthalenemethanol, decahydro-à,à,4a-trimethyl-8-methylene-, [2R-(2à,4aá,8aà)]-	222	C_15_H_26_O	4.90	Leaves, stem	GC-MS	[34]
223	820	à-Cadinol	222	C_15_H_26_O	1.35	Leaves, stem	GC-MS	[34]
224	619	2-Propen-1-ol, 2-bromo-, acetate	178	C_5_H_7_BrO_2_	0.138	Leaves, stem	GC-MS	[34]
225	783	Myo-Inositol, 2-C-methyl-	194	C_7_H_14_O_6_	3.22	Leaves, stem	GC-MS	[34]
226	743	2-Fluoro-6-trifluoromethylbenzoic acid, 4-cyanophenyl ester	309	C_15_H_7_F_4_NO_2_	0.03	Leaves, stem	GC-MS	[34]
227	740	Pyrimidine-2,4,6(1H,3H,5H)-trione, 1-benzyl-5-[1-(2-diethylaminoethylamino)propylidene]-	372	C_20_H_28_N4O_3_	0.17	Leaves, stem	GC-MS	[34]
228	877	1,2-Benzenedicarboxylic acid, dihexyl ester	334	C_20_H_30_O_4_	0.23	Leaves, stem	GC-MS	[34]
229	813	Dodecanoic acid	200	C_12_H_24_O_2_	0.34	Leaves, stem	GC-MS	[34]
230	661	3-(tert-Butyl)-4-methoxyphenyl 2,2,2-trifluoroacetate	276	C_13_H_15_F_3_O_3_	0.50	Leaves, stem	GC-MS	[34]
231	825	Kaur-16-ene	272	C_20_H_32_	2.82	Leaves, stem	GC-MS	[34]
232	844	Undecanoic acid, ethyl ester	214	C_13_H_26_O_2_	2.82	Leaves, stem	GC-MS	[34]
233	925	Heneicosane	296	C_21_H_44_	1.39	Leaves, stem	GC-MS	[34]
234	615	Carbonic acid, monoamide, N-(2,4-dimethoxyphenyl)-, propargyl ester	235	C_12_H_13_NO_4_	0.10	Leaves, stem	GC-MS	[34]
235	605	7-Amino-3-phenylcoumarin	237	C_15_H_11_NO_2_	0.34	Leaves, stem	GC-MS	[34]
236	582	4-tert-pentylphenol, trifluoroacetate ester	260	C_13_H_15_F_3_O_2_	0.04	Leaves, stem	GC-MS	[34]
237	520	Octadecanoic acid, 17-methyl-, methyl ester	312	C_20_H_40_O_2_	0.04	Leaves, stem	GC-MS	[34]
238	874	1-Iodo-2-methylundecane	296	C_12_H_25_I	1.31	Leaves, stem	GC-MS	[34]
239 240	646	4H,5H-Pyrano(4,3-b)pyran-4,5-dione, 2,3-dihydro-3-à-hydroxy-2-à-methyl-7-propenyl-	236	C_12_H_12_O_5_	3.34	Leaves, stem	GC-MS	[34]
241	591	9,10-Anthracenedibutanol, 9,10-dihydro-	324	C_22_H_28_O_2_	0.16	Leaves, stem	GC-MS	[34]
242	771	Acetic acid, [2-[(2-propenylamino)carbonyl]phenoxy]-	235	C_12_H_13_NO_4_	0.07	Leaves, stem	GC-MS	[34]
243	629	1-Butanone, 1,10-(2,4,6-trihydroxy-m-phenylene)di-	266	C_14_H_18_O_5_	0.35	Leaves, stem	GC-MS	[34]
244	638	(Z)-8-(but-3-yn-1-yl)-5-(pent-2-en-4-yn-1-yl)octahydroindolizine	241	C_17_H_23_N	0.10	Leaves, stem	GC-MS	[34]
245	709	Hexasiloxane, tetradecamethyl-	458	C_14_H_42_O5Si_6_	0.28	Leaves, stem	GC-MS	[34]
246	654	Lupulon	414	C_26_H_38_O_4_	0.46–2.28	Leaves, stem	GC-MS	[34,55]
247	1150	Menthone	154.25	C_10_H_18_O	1.44	Leaves	GC-MS	[46]
248	1245	Citronellol	156.27	C_10_H_20_O	2.88	Leaves	GC-MS	[46]
249	1275	Citronellyl formate	184.27	C_11_H_20_O_2_	2.34	Leaves	GC-MS	[46]
250	1276	Geraniol	154.25	C_10_H_18_O	1.60	Leaves	GC-MS	[46]
251	1590, 1593	Viridiflorol	222.37	C_15_H_26_O	0.35–16.8	Leaves, stem	GC-MS	[8,46]
252	-	Pentane, 2,2,4,4-tetramethyl-	128	C_9_H_20_	1.97	Leaves, stem	GC-MS	[55]
253	-	2,7-Octanedione	142	C_8_H_14_O_2_	2.08	Leaves, stem	GC-MS	[55]
254	-	Propane, 1,1-diethoxy-2-methyl-	146	C_8_H_18_O_2_	0.84	Leaves, stem	GC-MS	[55]
255	-	2-Pentanone, 5,5-diethoxy-	174	C_9_H_18_O_3_		Leaves, stem	GC-MS	[55]
256	-	à-Cubebene	204	C_15_H_24_	2.05	Leaves, stem	GC-MS	[55]
257	-	11,11-Dimethyl-4,8-dimethylenebicyclo[7.2.0] undecan-3-ol	220	C_15_H_24_O	1.70	Leaves, stem	GC-MS	[55]
258	-	trans-Farnesol	222	C_15_H_26_O	1.82	Leaves, stem	GC-MS	[55]
259	-	Tridecanoic acid, methyl ester	228	C_14_H_28_O_2_	0.70	Leaves, stem	GC-MS	[55]
260	-	Oxalic acid, allyl nonyl ester	256	C_14_H_24_O_4_	8.13	Leaves, stem	GC-MS	[55]
261	-	Gerany-P-cymene	270	C_20_H_30_	1.30	Leaves, stem	GC-MS	[55]
262	-	Linoleic acid	280	C_18_H_32_O_2_	9.16	Leaves, stem	GC-MS	[55]
263	-	Oleic acid amide	281	C_18_H_35_NO	11.33	Leaves, stem	GC-MS	[55]
264	-	Butyl palmitate	312	C_20_H_40_O_2_	2.00	Leaves, stem	GC-MS	[55]
265	-	Hexanedioic acid, bis(2-ethylhexyl) ester	370	C_22_H_42_O_4_	2.99	Leaves, stem	GC-MS	[55]
266	-	Heptacosane	380	C_27_H_56_	1.82	Leaves, stem	GC-MS	[55]
267	-	Stigmasterol	412	C_29_H_48_O	4.36	Leaves, stem	GC-MS	[55]
268	-	Heptacosane, 1-chloro-	414	C_27_H_55_Cl	2.13	Leaves, stem	GC-MS	[55]
269	-	á-Sitosterol	414	C_29_H_50_O	4.282	Leaves, stem	GC-MS	[55]
270	-	4,5-Di-O-caffeoylquinic acid	516.40	C_25_H_24_O_12_	-	Leaves, stem	GC-MS	[55]
271	1644.2	10,10-Dimethyl-2,6-dimethylenebicyclo [7.2.0]undecan-5β-ol	220.35	C_15_H_24_O	0.6, 0.2	Leaves, stem	GC-MS	[8]
272	-	4,5-dimethyl-11-methylenetricyclo [7.2.1.0 (4.9)]dodecane	222.37	C_15_H_26_O	0.1, 5.5	Leaves, stem	GC-MS	[8]
273	1427.0	Junipene	204.35	C_15_H_24_	0.2	Leaves, stem	GC-MS	[8]
274	1017.61	Para-cymene	134.22	C_10_H_14_	0.5	Leaves, stem	GC-MS	[8]
275	1028.75	M-cymene	134.22	C_10_H_14_	0.2	Leaves, stem	GC-MS	[8]
276	785.29	Toluene	92.14	C_6_H_5_CH_3_	0.2, 0.4	Leaves, stem	GC-MS	[8]
277	852.1	Para-xylene	106.16	C_6_H_4_(CH_3_)_2_	0.8	Leaves, stem	GC-MS	[8]
278	848.4	M-xylene	106.16	C_6_H_4_(CH_3_)_2_	1.9, 0.8	Leaves, stem	GC-MS	[8]
279	909.1	Xylene	106.16	C_6_H_4_(CH_3_)_2_	1.4	Leaves, stem	GC-MS	[8]
278	850.6	Benzene, ethyl-	106.16	C_8_H10	0.2, 0.4	Leaves, stem	GC-MS	[8]
279	848.4	Benzene, 1,4-dimethyl-	110.19	C_13_H_20_	Traces	Leaves, stem	GC-MS	[8]
280	852.1	Benzene, 1,3-dimethyl-	106.17	C_8_H_10_	1.9	Leaves, stem	GC-MS	[8]
281	1124.7	Benzene, 1,2,4,5-tetramethyl-	134.22	C_10_H_14_	0.1, 0.8	Leaves, stem	GC-MS	[8]
282	1129	Benzene, 1,2,3,5-tetramethyl-	134.22	C_10_H_14_	0.9	Leaves, stem	GC-MS	[8]
283	1616.2	Benzene, 1,1^′^-oxybis-	170.21	C_12_H_10_O	0.2–1.8	Leaves, stem	GC-MS	[8]
284	680.4	Cyclopentane, 1,3-dimethyl-, *cis*-	98.19	C_7_H_14_	4.0	Leaves, stem	GC-MS	[8]
285	948.55	α-Pinene oxide	152.23	C_10_H_16_O	0.3	Leaves, stem	GC-MS	[8]
286	2038	Humulene oxide	220.35	C_15_H_24_O	0.2	Leaves, stem	GC-MS	[8]
287	1192	Octen-1-ol, acetate	170.25	C_10_H_18_O_2_	0.5	Leaves, stem	GC-MS	[8]
288	1029	Benzene, 1-methyl-2-(1-methylethyl)-	160.25	C_12_H_16_	0.2–1.8	Leaves, stem	GC-MS	[8]
289	1005.5	Benzene, 1,2,4-trimethyl-	120.19	C_9_H_12_	0.1, 0.2	Leaves, stem	GC-MS	[8]
290	1070.8	Benzene, 1-ethyl-2,4-dimethyl-	134.22	C_10_H_14_	1.3	Leaves, stem	GC-MS	[8]
291	1135	Neoalloocimene	136.23	C_10_H_16_	0.2	Leaves, stem	GC-MS	[8]
292		Trans-1-(p-methoxyphenyl)-2,3,4-trimethyl-1-cyclobutene	202.30	C_14_H_18_O	0.4	Leaves. stem	GC-MS	[8]
293	1401.0	Methyl eugenol	178.23	C_11_H_14_O_2_	0.2–1.4	Leaves. stem	GC-MS	[8]
294	-	α-Zingiberene	204.35	C_15_H_24_	1.3, 3.2	Leaves. stem	GC-MS	[8]
295	1505	α-Selinene	204.35	C_15_H_24_	0.04–1.7	Leaves. stem	GC-MS	[8]
296	-	Cycloisolongifolene	204.35	C_15_H_24_	0.4, 5	Leaves. stem	GC-MS	[8]
297	-	(5 R)-2-cyano-5,9-dimethyldeca-2,8-dienenitrile	202.30	C_13_H_18_N_2_	0.3	Leaves. stem	GC-MS	[8]
298	-	6,10,11,11-tetramethyl-tricyclo [6.3.0.1 (2,3)]undec-1 (7)ene	204.35	C_15_H_24_	0.8, 2	Leaves. stem	GC-MS	[8]
299	1635	Alloaromadendrene	204.35	C_15_H_24_	0.7–1.7	Leaves. stem	GC-MS	[8]
300	1723	*Trans*-β-farnesene	204.35	C_15_H_24_	0.4–0.7	Leaves. stem	GC-MS	[8]
301	1479	γ-Gurjunene	204.35	C_15_H_24_	0.4, 0.6	Leaves. stem	GC-MS	[8]
302	1499.0	α-Muurolene	204.35	C_15_H_24_	0.4, 0.9	Leaves. stem	GC-MS	[8]
303	1503	Helminthogermacrene	204.35	C_15_H_24_	0.6	Leaves. stem	GC-MS	[8]
304	1545	Selina-3,7 (11)-diene	204.35	C_15_H_24_	1.1	Leaves. stem	GC-MS	[8]
305	1526	1 S,*Cis*-Calamenene	202.34	C_15_H_22_	0.2–0.9	Leaves. stem	GC-MS	[8]
306	1472	12-Nor-caryophyll-5-en-2-on	206.32	C_14_H_22_O	1.0	Leaves, stem	GC-MS	[8]
307	1515	Naphthalene, 1,2,3,4,4a,7-hexahydro-1,6-dimethyl-4-(1-methylethyl)-	204.35	C_15_H_24_	1.3, 0.4	Leaves, stem	GC-MS	[8]
308	1579	Globulol	222.37	C_15_H_26_O	0.4–2.5	Leaves, stem	GC-MS	[8]
309	1575.0	(+) Spathulenol	220.35	C_15_H_24_O	0.1–0.7	Leaves, stem	GC-MS	[8]
310	1640	T-Muurolol	222.37	C_15_H_26_O	0.3, 0.8	Leaves, stem	GC-MS	[8]
**311**	1656	Pogostol	222.37	C_15_H_26_O	1.1, 2.9	Leaves, stem	GC-MS	[8]
312	1772	1,4-dimethyl-7-(1-methylethyl)azulene	198.30	C_15_H_18_	0.2, 0.8	Leaves, stem	GC-MS	[8]
313	1844	2-Pentadecanone, 6,10,14-trimethyl-	268.48	C_18_H_36_O	0.2, 0.5	Leaves, stem	GC-MS	[8]
314	1311	Azulene	128.17	C_10_H_8_	0.3, 1.5	Leaves, stem	GC-MS	[8]
315	1282	Heptenyl acetate	158.24	C_9_H_18_O_2_	0.1	Aerial parts	GC-MS	[52]
316	1391	(Z)-3-Hexen-1-ol	100.16	C_6_H_12_O	0.1	Aerial parts	GC-MS	[52]
317	1413	Rosefuran	150.22	C_10_H_14_O	Traces	Aerial parts	GC-MS	[52]
318	1429	Perillen	150.22	C_10_H_14_O	Traces	Aerial parts	GC-MS	[52]
319	1468	*trans*-1,2-Limonene epoxide	152.23	C_10_H_16_O	0.1	Aerial parts	GC-MS	[52]
320	1568	1-Methyl-4-acetyl-cyclohex-1-ene	138.21	C_9_H_14_O	Traces	Aerial parts	GC-MS	[52]
321	1594	trans-β-Bergamotene	204.35	C_15_H_24_	0.8, 0.6	Aerial parts	GC-MS	[52]
322	1632	Selina-5,11-diene	204.35	C_15_H_24_	0.8, 0.9	Aerial parts	GC-MS	[52]
323	1751	Carvone	150.22	C_10_H_14_O	0.4	Aerial parts	GC-MS	[52]
324	1758	*cis*-Piperitol	154.25	C_10_H_18_O	0.1	Aerial parts	GC-MS	[52]
325	1782	*cis*-Carvyl acetate	194.27	C_12_H_18_O_2_	0.1, 0.2	Aerial parts	GC-MS	[52]
326	1796	Selina-3,7-(11)-diene	204.35	C_15_H_24_	1.1, 1.7	Aerial parts	GC-MS	[52]
327	1819	2-Phenyl ethyl acetate	164.20	C_10_H_12_O_2_	0.1	Aerial parts	GC-MS	[52]
328	1868	(E)-Geranyl acetate	196.29	C_12_H_20_O_2_	Traces	Aerial parts	GC-MS	[52]
329	2001	Isocaryophyllene oxide	220.35	C_15_H_24_O	0.1, 1.3	Aerial parts	GC-MS	[52]
330	2045	Humulene epoxide-I	220.35	C_15_H_24_O	0.5, 0.2	Aerial parts	GC-MS	[52]
331	2071	Humulene epoxide-II	220.35	C_15_H_24_O	1.3, 1.7	Aerial parts	GC-MS	[52]
332	2081	Humulene epoxide-III	220.35	C_15_H_24_O	0.1	Aerial parts	GC-MS	[52]
333	2184	*cis*-*p*-Menth-3-en-1,2-diol	170.25	C_10_H_18_O_2_	0.1	Aerial parts	GC-MS	[52]
334	2238	Clovenol	222.37	C_15_H_26_O	0.1	Aerial parts	GC-MS	[52]
335	2269	Porosadienol	222.37	C_15_H_26_O	0.3	Aerial parts	GC-MS	[52]
336	2308	9-Geranyl-*p*-cymene	284.4	C_20_H_28_O	0.7, 1.1	Aerial parts	GC-MS	[52]
335	2390	10-Hydroxy calamenene	218.34	C_15_H_22_O	0.2	Aerial parts	GC-MS	[52]
336	1494	epi-Cubebol	220.35	C_15_H_26_O	9.0	Aerial parts	GC-MS	[54]
337	1595	Octadienyl tiglate, *2E*, *4E*-	208.30	C_13_H_20_O_2_	2.1	Aerial parts	GC-MS	[54]
338	1636	β-Acorenol	222.37	C_15_H_26_O	0.5	Aerial parts	GC-MS	[54]
339	1660	Selin-11-en-4-ol	222.37	C_15_H_26_O	0.6	Aerial parts	GC-MS	[54]
340	1670	14-Hydroxy-9-epi-(E)-caryophyllene	220.35	C_15_H_24_O	5.0	Aerial parts	GC-MS	[54]
345	1685	epi-α-Bisabolol	222.37	C_15_H_26_O	5.6	Aerial parts	GC-MS	[54]
346	1713	14-Hydroxy- -humulene 2,4b-dimethyl-8-methylene-2-	220.35	C_15_H_24_O	1.4	Aerial parts	GC-MS	[54]
347	-	2,7-dimethyl-2,6-Octadiene	138.21	C_9_H_14_O	1.39	Leaves, stem	GC-MS	[34]
348	-	Acridine-9-carbaldehyde	207.23	C_14_H_9_NO	3.31	Leaves, stem	GC-MS	[34]
349	-	3,5-Dimethylcyclohex-1-ene-4-carboxaldehyde	138.21	C_9_H_14_O	4.91	Leaves, stem	GC-MS	[34]
350	-	Cedren-13-ol	220.35	C_15_H_24_O	1.59	Leaves, stem	GC-MS	[34]
351	-	Heptadecyl-oxirane	282.5	C_19_H_38_O	1.91	Leaves, stem	GC-MS	[34]
352	-	3a,7a-Dimethyl-hexahydro-2(3H)-Benzofuranone	168.236	C_10_H_16_O_2_	3.21	Leaves, stem	GC-MS	[34]
353	-	2-Ethyl-1,4-dimethyl-benzene	134.22	C_10_H_14_	2.26	Leaves, stem	GC-MS	[34]
354	-	6,10,14-trimethyl-2-pentadecanone	268.5	C_18_H_36_O	3.06	Leaves, stem	GC-MS	[34]
355	-	3-phenyl-3-methylbutanoic acid methyl ester	192.25	C_12_H_16_O_2_	1.31	Leaves, stem	GC-MS	[34]
356	-	Hexadecanoic acid methyl ester	270.45	C_17_H_34_O_2_	1.77	Leaves, stem	GC-MS	[34]
357	-	3-Methyl-N-naphthalen-1-yl-benzamide	261.32	C_18_H_15_NO	2.16	Leaves, stem	GC-MS	[34]
358	-	9,15-Octadecadienoic acid methyl ester	294.47	C_19_H_34_O_2_	2.49	Leaves, stem	GC-MS	[34]
359	-	12-Octadecenoic acid methyl ester	296.5	C_19_H_36_O_2_	2.41	Leaves, stem	GC-MS	[34]
360	-	2-(2-octenyl)-cyclopentanone	194.31	C_13_H_22_O	1.20	Leaves, stem	GC-MS	[34]
361	-	7-Tetradecyne	196.37	C_14_H_28_	1.06	Leaves, stem	GC-MS	[34]
362	-	9,12,15-Octadecatrienoic acid ethyl ester	306.48	C_20_H_34_O	1.38	Leaves, stem	GC-MS	[34]
363	-	3,7,11,16-tetramethyl-hexadeca-2,6,10,14-tetraen-1-ol	290.50	C_20_H_34_O	4.30	Leaves, stem	GC-MS	[34]
364	-	2-Nerolidol	222.37	C_15_H_26_O	3.39	Leaves, stem	GC-MS	[34]
365	-	(−)-Alloaromadendrene	204.35	C_15_H_24_	1.39	Leaves, stem	GC-MS	[34]
366	-	Isoaromadendrene epoxide	220.35	C_15_H_24_O	2.75	Leaves, stem	GC-MS	[34]
367	-	(+)-p-Mentha-2,8-diene	136.23	C_10_H_16_	1.29	Leaves, stem	GC-MS	[34]
368	-	4 Methylene-2,8,8 trimethyl-2-vinyl-bicyclo nonane	204.35	C_15_H_24_	1.22	Leaves, stem	GC-MS	[34]
379	-	6-(p-Tolyl)-2-methyl-2-heptenol	218.33	C_15_H_22_O	1.15	Leaves, stem	GC-MS	[34]
378	-	5-Ethyl-m-xylene	134.22	C_10_H_14_	1.15	Leaves, stem	GC-MS	[34]
379	-	Phthalic acid mono-2-ethylhexyl ester	278.34	C_16_H_22_O_4_	6.75	Leaves, stem	GC-MS	[34]
380	-	Phthalic acid, butyl pent-2-en-4-yn-1-yl ester	286.32	C_17_H_18_O_4_	6.98	Leaves, stem	GC-MS	[34]
381	-	Docosanoic acid methyl ester	354.61	C_23_H_46_O	11.44	Leaves, stem	GC-MS	[34]
382	-	Dodecanal dimethyl acetal	230.39	C_14_H_30_O_2_	3.16	Leaves, stem	GC-MS	[34]
383	-	Nonadecanoic acid, ethyl ester	326.56	C_21_H_42_O_2_	1.21	Leaves, stem	GC-MS	[34]
384	-	Z,E-3,13-Octadecadien-1-ol	266.46	C_18_H_34_O	6.28	Leaves, stem	GC-MS	[34]
385	-	Heneicosane	296.58	C_21_H_44_	2.83	Leaves, stem	GC-MS	[34]
386	-	1-(2-Isopropyl-5-methylcyclopentyl) ethanone	168.28	C_11_H_20_O	2.13	Leaves, stem	GC-MS	[34]
387	-	1,22-Docosanediol	202.40	C_12_H_25_SH	15.06	Leaves, stem	GC-MS	[34]

RI: Retention indices, MW: Molecular weight, MF: Molecular formula, GC–MS: Gas chromatography–mass spectrometry, IR: Infrared Spectroscopy, MS: Mass Spectrometry.

## Data Availability

Not applicable as no new data was created as it is a comprehensive review article.

## Abbreviations

The following abbreviations are used in this manuscript:

TB	Tuberculosis
GC-MS	Gas chromatography–mass spectrometry
RI	Retention indices
MW	Molecular weight
MF	Molecular formula
IR	Infrared Spectroscopy
INT	Iodonitrotetrazolium violet
MIC	Minimal inhibitory concentration
FIC	Fractional inhibitory concentrations
MBC	Minimum bactericidal concentration
DPPH	2,2-diphenyl-1-picrylhydrazyl
H-ORAC	Hydrophilic oxygen radical absorbance capacity
L-ORAC	Lipophilic oxygen radical absorbance capacity
ABTS	2,2′-azino-bis(3-ethylbenzothiazoline-6-sulfonic acid)
FRAP	Ferric Reducing Antioxidant Power
ORAC	Oxygen radical absorbance capacity
DMSO	Dimethyl sulfoxide
IL	Interleukin
